# Supplementary material for: Leveraging functional genomic annotations and genome coverage to improve polygenic prediction of complex traits within and between ancestries
Source: Nat Genet. 2024 Apr 30;56(5):767–77. doi: 10.1038/s41588-024-01704-y (PMC11096109; doi:10.1038/s41588-024-01704-y)
Supplement: Supplementary file 1 — Supplementary Note and Supplementary Figures 1–17. [file 41588_2024_1704_MOESM1_ESM.pdf]

nature genetics

Article https://doi.org/10.1038/s41588-024-01704-y

Leveraging functional genomic annotations and genome coverage to improve polygenic prediction of complex traits within and between ancestries

In the format provided by the authors and unedited

Table of Contents

Supplementary Note 2

1. Summary-data-based low-rank model 2

2. Advantages of using low-rank model 4

3. Estimation of residual variance helps to improve model robustness 5

4. Low-rank model does not disturb Bayesian learning on the annotation effects 6

5. Alternative parameterization for π 7

6. Scaling the SNP marginal effect estimates 8

7. Estimation of SNP-based heritability and per-SNP heritability enrichment for each annotation 9

8. MCMC sampling scheme 10

9. Calibrating parameters for the low-rank model 14

10. Tuning for the optimal eigenvalue cut-off 14

11. Violation of model assumptions 17

12. Sensitivity analysis in simulation for SBayesRC 17

13. Simulation under the S-LDSC/MegaPRS model 18

14. Difference between the LDSC/MegaPRS model and SBayesRC model 18

15. Comparison between SBayesRC and MegaPRS 19

16. Other factors affecting accuracy of prediction leveraging functional annotations 20

17. Summary data imputation 22

18. Running settings for the cross-validation in the UKB 22

19. Running settings for trans-ancestry prediction 23

20. Reason for using cross-validation in the UKB analysis 24

21. Acknowledgements 25

Supplementary Figures 27

Reference 44

Supplementary Note

1. Summary-data-based low-rank model

To simultaneously analyse all common variants, SBayesRC utilises a low-rank model based on the eigen-decomposition on quasi-independent LD blocks in the human genome[1]. In each LD block k, the summary-data-based model (as used in SBayesR) is transformed into a new model where the joint effects of m_k SNPs are fitted to q_k linear combinations of marginal SNP effects (instead of m_k marginal SNP effects), with q_k being the number of top principal components (PCs) that collectively explain at least a given proportion (ρ) of variance in the LD matrix (**Extended Data Fig. 1a**). In this model, the dimension of the system of equations is $q_k \times m_k$, with q_k much smaller than m_k, which reduces memory consumption and speeds up computation significantly. For instance, when $\rho = 99.5\%$, $q_k/m_k \approx 0.2$ on average across LD blocks for the 7.4 million common SNPs used in this study (**Supplementary Fig. 1**). Essentially, the low-rank model refines the signals in GWAS summary statistics by collapsing information from SNPs in high LD. In addition to the computational advantages, the low-rank transformation enhances model robustness by 1) removing small PCs that are subject to LD differences between GWAS and reference samples, and 2) enabling a Gibbs sampling algorithm to directly estimate the residual variance, a nuisance parameter accommodating remaining LD variations and potential errors in GWAS summary data.

We derive a summary-data-based low-rank model from a general form of individual-level linear regression. Consider model

$$\mathbf{y} = \mathbf{X}\boldsymbol{\beta} + \mathbf{e} \quad (1)$$

where $\mathbf{y}$ is the vector of trait phenotypes adjusted for covariates, such as sex, age and principal components (PCs), $\mathbf{X}$ is the genotype matrix of m SNPs standardised to have column mean zero and variance one, $\boldsymbol{\beta}$ is the vector of true SNP effects, and $\mathbf{e}$ are the residuals with $Var(\mathbf{e}) = \mathbf{I}\sigma_e^2$. Let N be the sample size. Multiplying both sides of the equation by $\frac{1}{N}\mathbf{X}'$ gives

$$\frac{1}{N}\mathbf{X}'\mathbf{y} = \frac{1}{N}\mathbf{X}'\mathbf{X}\boldsymbol{\beta} + \frac{1}{N}\mathbf{X}'\mathbf{e}$$

The left-hand side is the GWAS marginal effect estimates $\mathbf{b}$. Let $\mathbf{R} = \frac{1}{N}\mathbf{X}'\mathbf{X}$ be the LD correlation matrix. Then, we have

$$\mathbf{b} = \mathbf{R}\boldsymbol{\beta} + \frac{1}{N}\mathbf{X}'\mathbf{e}$$

This is the summary-data-based model underlying many methods. Of note, in this model, the residuals have a variance-covariance structure proportional to the LD matrix, i.e., $Var\left(\frac{1}{N}\mathbf{X}'\mathbf{e}\right) = \frac{1}{N}\mathbf{R}\sigma_e^2$. It is often neither feasible nor necessary to compute the whole-genome LD matrix. Alternatively, we compute **R** for each of the LD blocks that are found to be approximately independent in the human population. In this case, the genome-wide LD matrix is a block-diagonal matrix with blocks defined by LD blocks. The eigen-decomposition of $\mathbf{R}_i$ for block *i*, which can be performed independently and in parallel between block, is (the subscript is ignored for simplicity in notation)

$$\mathbf{R} = \mathbf{U\Lambda U}'$$

where **U** is the matrix of eigenvectors and $\mathbf{\Lambda}$ is the diagonal matrix of eigenvalues. Substitution of **R** in the equation above gives

$$\mathbf{b} = \mathbf{U\Lambda U}'\boldsymbol{\beta} + \frac{1}{N}\mathbf{X}'\mathbf{e}$$

Multiplying both sides by $\mathbf{\Lambda}^{-\frac{1}{2}}\mathbf{U}'$ gives

$$\mathbf{\Lambda}^{-\frac{1}{2}}\mathbf{U}'\mathbf{b} = \mathbf{\Lambda}^{\frac{1}{2}}\mathbf{U}'\boldsymbol{\beta} + \frac{1}{N}\mathbf{\Lambda}^{-\frac{1}{2}}\mathbf{U}'\mathbf{X}'\mathbf{e}$$

or simply,

$$\mathbf{w} = \mathbf{Q}\boldsymbol{\beta} + \boldsymbol{\epsilon} \quad (2)$$

where $\mathbf{w} = \mathbf{\Lambda}^{-\frac{1}{2}}\mathbf{U}'\mathbf{b}$ is a linear combination of marginal SNP effect estimates, $\mathbf{Q} = \mathbf{\Lambda}^{\frac{1}{2}}\mathbf{U}'$ is the new coefficient matrix, and the new residuals $\boldsymbol{\epsilon} = \frac{1}{N}\mathbf{\Lambda}^{-\frac{1}{2}}\mathbf{U}'\mathbf{X}'\mathbf{e}$ are independently and identically distributed, i.e.,

$$Var(\boldsymbol{\epsilon}) = \frac{1}{N}\mathbf{\Lambda}^{-\frac{1}{2}}\mathbf{U}'\mathbf{X}'\mathbf{X}\mathbf{U}\mathbf{\Lambda}^{-\frac{1}{2}}\frac{1}{N}$$
$$= \frac{1}{N}\mathbf{I}\sigma_\epsilon^2$$

Due to LD between SNPs and limited sample size, the LD matrix estimated from a reference sample is often rank deficient. In this case, a number of eigenvalues are zero. Additionally, small eigenvalues are subject to sampling variation in LD between GWAS and LD reference samples. To this end, we partition $\mathbf{\Lambda}$ into

$$\mathbf{\Lambda} = \begin{bmatrix} \mathbf{\Lambda}_q & \mathbf{0} \\ \mathbf{0} & \mathbf{\Lambda}_0 \end{bmatrix}$$

where $\mathbf{\Lambda}_q$ contains *q* eigenvalues in descending order that cumulatively explain at least a given proportion (ρ) of variance in LD, i.e., $\rho = \frac{\sum_{i=1}^{q}\Lambda_i}{\sum_{i=1}^{m}\Lambda_i}$ where Λ_i is the ith nonzero

eigenvalue, and $\mathbf{\Lambda}_0$ contains remaining eigenvalues including zeros. Then the model can be written as

$$\begin{bmatrix}\mathbf{w}_q\\ \mathbf{w}_0\end{bmatrix} = \begin{bmatrix}\mathbf{Q}_q\\ \mathbf{Q}_0\end{bmatrix}\boldsymbol{\beta} + \begin{bmatrix}\boldsymbol{\epsilon}_q\\ \boldsymbol{\epsilon}_0\end{bmatrix}$$

To remove the noise in LD, we discard the equations for $\mathbf{w}_0$, resulting in a low-rank model:

$$\mathbf{w}_q = \mathbf{Q}_q\boldsymbol{\beta} + \boldsymbol{\epsilon}_q$$

where $\mathbf{Q}_q$ has a dimension of $q \times m$ with $q \ll m$. In essence, the true SNP effects are fitted to a smaller number of effective data points rather than the observed data points that are highly correlated to each other. This model is general and can be applied with different assumptions on the distribution of SNP effects $\boldsymbol{\beta}$.

2. Advantages of using low-rank model

We proposed a rank reduction approach to account for correlations between marginal effects, allowing the joint effects of genome-wide SNPs to be fitted to a set of observables with independent residuals. There are several advantages of using this low-rank model. First, it substantially improved the model robustness. We showed that our method is robust to LD differences between GWAS and reference samples, as well as per-SNP sample size variations resulted from the meta-analysis between cohorts with different genotyping platforms. As shown in the real trait analysis (**Fig. 3b**, **Fig. 4c,d,** and **Extended Data Fig. 7**), SBayesRC consistently yielded robust prediction accuracy, when using an external LD reference independent of the GWAS sample, outperforming other methods. We recommend using in-sample LD for improved prediction accuracy, but when this is not possible, SBayesRC is expected to exhibit greater robustness compared to other methods. The low ranking is not only due to the elimination of many small eigenvalues/eigenvectors in each LD block, which are subject to high sampling variation in LD, but also because the independence of transformed summary statistics enables the sampling of block-wise residual variance, which introduces a mechanism to manage the convergence issue caused by violations of model assumptions (**Supplementary Note 3**). It has been found that SNP effect sizes can blow up during MCMC when the model fails to converge. In this case, the sampled values of residual variances would be large, causing the SNP effect sizes to shrink back toward zero, thereby preventing convergence failure. Second, the low-rank model substantially improved the computational efficiency, which allows us to fit a large number of SNPs with only a small fraction of computation resource compared to the original model. In theory, SBayesRC is scalable to fit variants at the sequence level via calibrating the required proportion of

variance in LD explained by the selected eigenvalues/eigenvectors, making it a powerful tool to analyse the incoming large-scale whole-genome sequence data. Third, this low-rank model depicts a general framework that can be applied with different priors, as used in other methods.

3. Estimation of residual variance helps to improve model robustness

In the summary-data-based model, Eq (1), $Var(\boldsymbol{\varepsilon}) = \frac{1}{N}\mathbf{R}\sigma_\epsilon^2$. It is often assumed that $\sigma_\epsilon^2 \approx \sigma_y^2$ given a negligible proportion of variance explained by a single SNP, and further $\sigma_\epsilon^2 \approx 1$ assuming a unit phenotypic variance for the trait. It is possible, however, that $\sigma_\epsilon^2 > 1$ if there exist large LD differences between GWAS and LD reference samples. This is because using summary statistics from GWAS and inaccurate LD data from a reference is analogous to using estimated genotype data with noise in the fitted model for GWAS:

$$\begin{aligned}\mathbf{y} &= \mathbf{1}\mu + \widehat{\mathbf{X}}\hat{b} + \mathbf{e}\\ &= \mathbf{1}\mu + (\mathbf{X} + \boldsymbol{\Delta})\hat{b} + \mathbf{e}\\ &= \mathbf{1}\mu + \mathbf{X}\hat{b} + \left(\boldsymbol{\Delta}\hat{b} + \mathbf{e}\right)\\ &= \mathbf{1}\mu + \mathbf{X}\hat{b} + \mathbf{e}^*\end{aligned}$$

where $\widehat{\mathbf{X}}$ is the combination of genotypes used in GWAS ($\mathbf{X}$) and the differences to those observed from the reference ($\boldsymbol{\Delta}$), and $\hat{b}$ is the ordinary least squares estimate for the SNP effect. It can be seen that the new residual in the above model can have variance larger than the phenotypic variance when the noise in the genotype data is large, i.e., $Var\left(\Delta\hat{b} + \mathrm{e}\right) > \mathrm{Var}(y)$. Thus, it would be beneficial to estimate the residual variance from the data given the GWAS summary statistics and reference LD data.

In contrast to Eq (1), it is very straightforward to estimate the residual variance in the low-rank model, Eq (2), because the residuals are independently distributed, $Var(\boldsymbol{\epsilon}) = \frac{1}{N}\mathbf{I}\sigma_\epsilon^2$. The MCMC sampling process for the residual variance is shown as below. The large residual variance estimate will introduce a shrinkage mechanism to manage the potential convergence issue due to violation of model assumptions. It has been found that SNP effect sizes would blow up during MCMC when the model fails to converge. In this case, the sampled values of residual variances would be large if the SNP effect sizes tend to blow up, which will in turn shrink them back toward zero, preventing the failure in convergence.

4. Low-rank model does not disturb Bayesian learning on the annotation effects

An annotation-dependent prior is used in SBayesRC to better model the distribution of SNP effects and to learn both annotation parameters and SNP effects from the data. In SBayesR, SNP effects follow a mixture of normal distributions with different variances, including a point mass at zero, capturing various genetic architectures. However, it is independent of genomic features, such as LD and minor allele frequency (MAF) patterns and biological functions of different genomic regions. In SBayesRC, the probability parameters of the mixture distribution are estimated from a generalised linear model with individual SNP annotations as independent variables (**Extended Data Fig. 1b**). Both binary and quantitative annotations are accommodated, regardless of overlap between them. This approach describes the probability that SNPs are causal variants and the probability distribution of their effect sizes, better capturing the causal effects if the distributions of effect sizes truly differ between annotations. It is of note that the Bayesian learning machinery on the annotation effects is not disturbed by the low-rank reduction at the level of summary data.

The low-rank model is derived from the standard summary-data-based model, where *m* SNP marginal effects (**b**) are fitted with *m* SNP joint effects ($\boldsymbol{\beta}$):

$$\mathbf{b} = \mathbf{R}\boldsymbol{\beta} + \boldsymbol{\varepsilon}$$

with **R** being a $m \times m$ matrix and $\boldsymbol{\varepsilon}$ being mutually correlated with a variance-covariance matrix proportional to **R**. In each LD block, we perform eigen-decomposition on R such that

$$\mathbf{b} = \mathbf{U}\boldsymbol{\Lambda}\mathbf{U}'\boldsymbol{\beta} + \boldsymbol{\varepsilon} \quad (3)$$

where **U** and $\boldsymbol{\Lambda}$ are matrices of eigenvectors and eigenvalues, respectively. The low-rank model is constructed such that the dimension of observed data (**b** and **R**) is reduced but the dimension of parameters ($\boldsymbol{\beta}$) remains. In essence, it fits *q* observables with *m* SNP joint effects, where $q << m$ and the observables are correlated only through SNP joint effects but not residuals:

$$\mathbf{w} = \mathbf{Q}\boldsymbol{\beta} + \boldsymbol{\epsilon}$$

with **w** being a $q \times 1$ vector and **Q** being a $q \times m$ matrix. As shown in our simulation and real data analyses, the low-rank model approximates the standard model with high accuracy, when the choice of *q* is well calibrated, and has considerable improvement in computational efficiency and robustness. The functional annotations are incorporated into the prior distribution of β_j:

$$\beta_j \sim \sum_k \pi_{jk} N(0, \sigma_k^2)$$

$$f(\pi_{jk}) = \mathbf{A}_j \boldsymbol{\alpha}_k$$

with $\mathbf{A}_j$ being the observed annotation data matrix for SNP j and $\boldsymbol{\alpha}_k$ being the vector of unknown annotation effects estimated from the data. In this model, functional annotations describe the prior distribution of SNP joint effects, thereby contributing to the posterior estimation of SNP weights. It is a hierarchical Bayesian learning process for $\boldsymbol{\alpha}_k$ because $\boldsymbol{\alpha}_k$ connect to the data (**w** and **Q**) only through $\boldsymbol{\beta}$, which means that $\boldsymbol{\alpha}_k$ can be estimated in either the standard model (1) or the low-rank model (2) since both models have $\boldsymbol{\beta}$ as parameters. Note that an alternative reduction is to reduce the dimension of parameter space, that is, from Eq (3) we can have

$$\mathbf{b} = \mathbf{U}\boldsymbol{\beta}^* + \boldsymbol{\varepsilon}$$

where **U** is a $m \times q$ matrix and $\boldsymbol{\beta}^* = \boldsymbol{\Lambda}\mathbf{U}'\boldsymbol{\beta}$ becomes a $q \times 1$ vector that captures the principal component effects. In this case, the Bayesian machinery to learn about the different weights associated with functional annotations would not work because each element of $\boldsymbol{\beta}^*$ is not the SNP joint effect itself but a linear combination of SNP joint effects.

In summary, the Bayesian learning about functional annotations works in the low-rank model because the low rank approximation still captures >99% of the variance. However, it is expected that with a lower eigenvalue threshold (e.g., 50%), the performance would be compromised.

5. Alternative parameterization for $\boldsymbol{\pi}$

To remove the dependence between elements of $\boldsymbol{\pi}_j$ for each SNP, we employed an alternative parameterization for modelling membership probabilities and annotation effects. Let δ_j be the indicator for the mixture component membership for SNP j:

$$\delta_j = k \text{ with probability } \pi_{jk}; k = 1 \text{ to } 5$$

We define a conditional probability that the SNP effect belongs to the kth distribution given that it has passed the bar for the $(k\text{-}1)^{\text{th}}$ distribution as

$$p_{jk} = \Pr(\delta_j \geq k \mid \delta_j \geq k-1) \text{ for } k \geq 2$$

such that

$$\pi_{j1} = 1 - p_{j2}$$

$$\pi_{j2} = (1 - p_{j3}) p_{j2}$$

$$\pi_{j3} = (1 - p_{j4})p_{j3}p_{j2}$$
$$\pi_{j4} = (1 - p_{j5})p_{j4}p_{j3}p_{j2}$$
$$\pi_{j5} = p_{j5}p_{j4}p_{j3}p_{j2}$$

We then apply the generalised linear model to link p_{jk} with $\boldsymbol{\alpha}_k$, i.e.,

$$g(p_{jk}) = \mu_k + \sum_{c=1}^{C} A_{jc}\alpha_{kc}$$

In this parameterisation, all p_{jk} are independent, which means that $\boldsymbol{\alpha}_k$ can be sampled in parallel in each MCMC iteration, and α_{kc} can be sampled from its full conditional distribution using Gibbs sampling algorithm, following the algorithm of Albert and Chib[2].

Let z_{jk} be the indicator variable for whether the SNP effect can "climb" up to a higher distribution, i.e.,

$$z_{jk} \sim Bernoulli(p_{jk})$$

To allow Gibbs sampling, a probit link function is chosen, namely $g^{-1}(p_{jk}) = \Phi(p_{jk})$ where $\Phi(\cdot)$ is the cumulative density function (CDF) of the standard normal distribution. It has been shown that with an auxiliary variable l_{jk} defined as

$$z_{jk} = \begin{cases} 0, & l_{jk} > 0 \\ 1, & l_{jk} \leq 0 \end{cases}$$

a linear model can be constructed as

$$l_{jk} = \mu_k + \sum_{c=1}^{C} A_{jc}\alpha_{kc} + \varepsilon_{jk}$$

with $\varepsilon_{jk} \sim N(0,1)$. In this model, given a normal prior distribution, $\alpha_{kc} \sim N(0, \sigma^2_{\alpha_k})$, the full conditional distribution for α_{kc} is a univariate normal distribution, since α_{kc} is conditionally independent of $\boldsymbol{z}_k$ given $\boldsymbol{l}_k$. Given z_{jk} and $\boldsymbol{\alpha}_k$, the full conditional distribution for the latent variable l_{jk} is a truncated normal distribution. The Gibbs sampling procedure is described in the following section.

6. Scaling the SNP marginal effect estimates

The derivation for the summary-data-based model is based on the marginal effects in units of per standardized genotype (**b**). When the marginal effects were estimated from GWAS using genotypes at 0/1/2 scale (**b***), **b** can be estimated, in a scalar form, by

$$b_j = s_j b_j^* \text{ where } s_j = \sqrt{\frac{\sigma_y^2}{N_j \sigma_j^2 + \left(b_j^*\right)^2}}$$

where σ_y^2 is the phenotypic variance, N_j is the per-SNP sample size, and σ_j is the standard error for SNP j. If the trait phenotypes are not standardized, the phenotypic variance can be estimated by taking the median value of $2f_j(1 - f_j)\left[N_j \sigma_j^2 + \left(b_j^*\right)^2\right]$ across SNPs, where f_j is the allele frequency in the GWAS sample (ref[3,4]). The per-SNP sample size N_j can be replaced by the overall sample size *N*. Here, we assume $\sigma_y^2 = 1$, then

$$s_j = \sqrt{\frac{1}{N_j \sigma_j^2 + \left(b_j^*\right)^2}}$$

and will scale the joint effect estimate β_j back to the phenotypic scale using the same s_j so that this parsimonious assumption would not have an impact on the result (ref.[5,6]).

7. Estimation of SNP-based heritability and per-SNP heritability enrichment for each annotation

The total genetic variance is

$$\begin{aligned}
\sigma_g^2 &= \boldsymbol{\beta}'\mathbf{R}\boldsymbol{\beta} \\
&= \boldsymbol{\beta}'\mathbf{U}\boldsymbol{\Lambda}\mathbf{U}'\boldsymbol{\beta} \\
&= \boldsymbol{\beta}'\mathbf{U}\boldsymbol{\Lambda}^{\frac{1}{2}}\boldsymbol{\Lambda}^{\frac{1}{2}}\mathbf{U}'\boldsymbol{\beta} \\
&= \boldsymbol{\beta}'\mathbf{Q}\mathbf{Q}\boldsymbol{\beta} \\
&= \widehat{\mathbf{w}}'\widehat{\mathbf{w}}
\end{aligned}$$

We calculate this quantity in each of MCMC iterations given the sampled values of SNP effects $\boldsymbol{\beta}$. Assuming unit phenotypic variance, the SNP-based heritability $h_{\text{SNP}}^2 = \sigma_g^2$, estimated by the posterior mean of MCMC samples discarding the samples from the burn-in period.

For a binary annotation *c*, the total variance explained by the SNPs within the annotation is calculated as

$$\sigma_c^2 = \sum_{j=1}^{m_c} \beta_{jc}^2$$

where m_c is the number of SNPs within the annotation. The per-SNP heritability enrichment (θ_c) is then calculated as

$$\theta_c = \frac{\sigma_c^2}{m_c} \Big/ \frac{\sigma_g^2}{m}$$

For a quantitative annotation, the per-SNP heritability enrichment is calculated as the slope of the regression of β_{jc}^2 on the annotation value A_{jc}

$$\mathrm{E}[\boldsymbol{\beta}_c^2] = \mathbf{1}\mu_c + \mathbf{A_c}\omega_c$$

and $\theta_c = 1 + \omega_c$. Similarly, we compute θ_c in every iteration of MCMC and estimate by the posterior mean after burn-in.

8. MCMC sampling scheme

We use MCMC sampling to draw posterior inference on the model parameters. The joint distribution of data and all parameters in the low-rank model is

$$f(\mathbf{w}, \boldsymbol{\beta}, \boldsymbol{\pi}, \mathbf{z}, \boldsymbol{\alpha}, \boldsymbol{\sigma}_\alpha^2, \sigma_\epsilon^2) \propto (\sigma_\epsilon^2)^{-\frac{q}{2}} \exp\left\{-\frac{(\mathbf{w} - \mathbf{Q}\boldsymbol{\beta})'(\mathbf{w} - \mathbf{Q}\boldsymbol{\beta})}{2\frac{\sigma_\epsilon^2}{n}}\right\}$$

$$\times \prod_{j=1}^{m} \left\{\sum_{k=1}^{5} \pi_{jk} \left[\exp\left\{-\frac{\beta_j^2}{2\gamma_k\sigma_g^2}\right\}\right]\right\}$$

$$\times \prod_{k=2}^{5} \prod_{j=1}^{m} \Phi(\mu_k + \mathbf{A}_j'\boldsymbol{\alpha}_k)^{z_{jk}} [1 - \Phi(\mu_k + \mathbf{A}_j'\boldsymbol{\alpha}_k)]^{(1-z_{jk})}$$

$$\times \prod_{k=2}^{5} \prod_{c=1}^{C} (\sigma_{\alpha_c}^2)^{-\frac{1}{2}} \exp\left\{-\frac{\alpha_{kc}^2}{2\sigma_{\alpha_c}^2}\right\}$$

$$\times \prod_{k=2}^{5} (\sigma_{\alpha_c}^2)^{-\frac{2+v_\alpha}{2}} \exp\left\{-\frac{v_\alpha \tau_\alpha^2}{2\sigma_{\alpha_c}^2}\right\}$$

$$\times (\sigma_\epsilon^2)^{-\frac{2+v_\epsilon}{2}} \exp\left\{-\frac{v_\epsilon \tau_\epsilon^2}{2\sigma_\epsilon^2}\right\}$$

Suppose δ_j is the indicator variable to the distribution membership of β_j. The full conditional distribution for β_j is

$$f(\beta_j | \mathbf{w}, \boldsymbol{\beta}_{-j}, \delta_j, \sigma_g^2, \sigma_\epsilon^2)$$

$$\propto (\sigma_\epsilon^2)^{-\frac{q}{2}} \exp\left\{-\frac{(\mathbf{w} - \sum_{j'\neq j} \mathbf{Q}_{j'}\beta_{j'})'(\mathbf{w} - \sum_{j'\neq j} \mathbf{Q}_{j'}\beta_{j'})}{2\frac{\sigma_\epsilon^2}{n}}\right\} \exp\left\{-\frac{\beta_j^2}{2\gamma_k\sigma_g^2}\right\}$$

$$= N\left(\frac{r_j}{C_j}, \frac{\sigma_\epsilon^2}{C_j}\right)$$

where

$$r_j = \mathbf{Q}_j'\left(\mathbf{w} - \sum\nolimits_{j' \neq j} \mathbf{Q}_{j'}\beta_{j'}\right)$$
$$= \mathbf{Q}_j'\mathbf{e} + \beta_j$$
$$C_j = 1 + \frac{\sigma_\epsilon^2}{\gamma_k \sigma_g^2}$$

The full conditional distribution for δ_j is

$$\Pr(\delta_j = k|\mathbf{w}, \boldsymbol{\beta}, \sigma_g^2, \sigma_\epsilon^2) = \frac{f(\mathbf{w}|\delta_j = k, \boldsymbol{\beta}, \sigma_g^2, \sigma_\epsilon^2) f(\delta_j = k)}{\sum_{k'=1}^{5} f(\mathbf{w}|\delta_j = k', \boldsymbol{\beta}, \sigma_g^2, \sigma_\epsilon^2) f(\delta_j = k')}$$

where $f(\mathbf{w}|\delta_j = k, \boldsymbol{\beta}, \sigma_g^2, \sigma_\epsilon^2)$ is shown as above and $f(\delta_j = k) = \pi_k$.

As described above, $\boldsymbol{\pi}_j$ is a function of $\boldsymbol{p}_j$, and $\boldsymbol{p}_j$ is a linear model of $\boldsymbol{\alpha}_k$ through the probit link, i.e.,

$$p_{jk} = \Phi^{-1}(\mu_k + \mathbf{A}_j'\boldsymbol{\alpha}_k)$$

Here, we introduce another indicator variable z_{jk}, where

$$z_{jk} = 1 \text{ if } \delta_j = k \text{ for } k \geq 2$$

and a latent variable l_{jk}, for which the full conditional distribution is

$$l_{jk}|z_{jk}, \mu_k, \boldsymbol{\alpha}_k = \begin{cases} TN(\mu_k + \mathbf{A}_j'\boldsymbol{\alpha}_k, 1, 0, \infty), & \text{if } z_{jk} = 1 \\ TN(\mu_k + \mathbf{A}_j'\boldsymbol{\alpha}_k, 1, -\infty, 0), & \text{if } z_{jk} = 0 \end{cases}$$

Similar to the sampling of SNP effects, we use single-site Gibbs sampler to sample the annotation effects. The full conditional distribution for α_{kc} is

$$f(\alpha_{kc}|\boldsymbol{l}_k, \boldsymbol{\alpha}_{-kc}, \sigma_{\alpha_k}^2)$$
$$\propto \exp\left\{-\frac{1}{2}\left(\boldsymbol{l}_k - \sum\nolimits_{c' \neq c} \mathbf{A}_{c'}\alpha_{kc'}\right)'\left(\boldsymbol{l}_k - \sum\nolimits_{c' \neq c} \mathbf{A}_{c'}\alpha_{kc'}\right)\right\} \exp\left\{-\frac{\alpha_{kc}^2}{2\sigma_{\alpha_k}^2}\right\}$$
$$= N\left(\frac{r_{kc}}{C_{kc}}, \frac{1}{C_{kc}}\right)$$

where

$$r_{kc} = \mathbf{A}_c'\left(\boldsymbol{l}_k - \sum\nolimits_{j' \neq j} \mathbf{A}_{c'}\alpha_{kc'}\right)$$
$$C_{kc} = \mathbf{A}_c'\mathbf{A}_c + \frac{1}{\sigma_{\alpha_k}^2}$$

The full conditional distribution for $\sigma_{\alpha_k}^2$ is

$$f(\sigma^2_{\alpha_k}|\boldsymbol{\alpha}_k) \propto f(\boldsymbol{\alpha}_k|\sigma^2_{\alpha_k})f(\sigma^2_{\alpha_k})$$
$$\propto (\sigma^2_{\alpha_k})^{-\frac{C+\upsilon_\alpha+2}{2}} \exp\left\{-\frac{\boldsymbol{\alpha}'_k\boldsymbol{\alpha}_k + \upsilon_\alpha\tau^2_\alpha}{2\sigma^2_{\alpha_k}}\right\}$$
$$= \chi^{-2}(\tilde{\upsilon}_\alpha, \tilde{\tau}^2_\alpha)$$

where $\tilde{\upsilon}_\alpha = C + \upsilon_\alpha$ and $\tilde{\tau}^2_\alpha = (\boldsymbol{\alpha}'_k\boldsymbol{\alpha}_k + \upsilon_\alpha\tau^2_\alpha)/\tilde{\upsilon}_\alpha$.

The full conditional distribution for σ^2_ϵ is

$$f(\sigma^2_\epsilon|\mathbf{w},\boldsymbol{\beta}) \propto f(\mathbf{w}|\boldsymbol{\beta},\sigma^2_\epsilon)f(\sigma^2_\epsilon)$$
$$\propto (\sigma^2_\epsilon)^{-\frac{q}{2}} \exp\left\{-\frac{(\mathbf{w}-\sum_j \mathbf{Q}_j\beta_j)'(\mathbf{w}-\sum_j \mathbf{Q}_j\beta_j)}{2\frac{\sigma^2_\epsilon}{n}}\right\}(\sigma^2_\epsilon)^{-\frac{\upsilon_\epsilon+2}{2}} \exp\left\{-\frac{\upsilon_\epsilon\tau^2_\epsilon}{2\sigma^2_\epsilon}\right\}$$
$$\propto (\sigma^2_\epsilon)^{-\frac{q+\upsilon_\epsilon+2}{2}} \exp\left\{-\frac{\boldsymbol{\epsilon}'\boldsymbol{\epsilon} + \upsilon_\epsilon\tau^2_\epsilon}{2\sigma^2_\epsilon}\right\}$$
$$= \chi^{-2}(\tilde{\upsilon}_\epsilon, \tilde{\tau}^2_\epsilon)$$

where $\tilde{\upsilon}_\epsilon = q + \upsilon_\epsilon$ and $\tilde{\tau}^2_\epsilon = (\boldsymbol{\epsilon}'\boldsymbol{\epsilon} + \upsilon_\epsilon\tau^2_\epsilon)/\tilde{\upsilon}_\epsilon$.

Algorithm pseudo code

**SBayesRC algorithm**
**Input**: GWAS summary statistics, reference LD correlation matrix, functional annotation data
Scale the GWAS marginal effect estimate $b_j = s_j b_j^*$
Construct the low-rank model by performing eigen-decomposition on LD blocks
Initialize model parameters
**for** i :=1 **to** number of iterations **do**
⠀⠀**for** j := 1 **to** number of SNPs **do**
⠀⠀⠀⠀Calculate $r_j = \mathbf{Q}_j' \mathbf{w}_{corr} + \beta_j$
⠀⠀⠀⠀Calculate $C_j = 1 + \frac{\sigma_\epsilon^2}{\gamma_k \sigma_g^2}$ for each γ_k
⠀⠀⠀⠀Calculate the posterior probabilities of SNP effect distribution memberships and sample δ_j
⠀⠀⠀⠀Sample SNP effect β_j from its full conditional distribution $N\left(\frac{r_j}{C_j}, \frac{\sigma_\epsilon^2}{C_j}\right)$
⠀⠀⠀⠀Given the sampled value of β_j^{new}, adjust $\mathbf{w}_{corr}^{new} = \mathbf{w}_{corr}^{old} + \mathbf{Q}_j\left(\beta_j^{old} - \beta_j^{new}\right)$
⠀⠀⠀⠀Calculate indicator variables $\mathbf{z}_j$ given δ_j
⠀⠀**end**
⠀⠀**for** k := 2 **to** number of mixture distribution components **do**
⠀⠀⠀⠀**for** j := 1 **to** number of SNPs that passed the bar for current component **do**
⠀⠀⠀⠀⠀⠀Sample latent variable l_{jk} from a truncated normal distribution given z_{jk}
⠀⠀⠀⠀**end**
⠀⠀⠀⠀**for** c := 1 **to** number of annotations **do**
⠀⠀⠀⠀⠀⠀Sample annotation effect α_{kc} from its full conditional distribution given $\boldsymbol{l}_k$
⠀⠀⠀⠀**end**
⠀⠀⠀⠀Sample annotation effect variance $\sigma_{\alpha_k}^2$ from its full conditional distribution given $\boldsymbol{\alpha}_k$
⠀⠀**end**
⠀⠀Calculate $\hat{\mathbf{w}} = \mathbf{Q}\boldsymbol{\beta}$ and total genetic variance $\sigma_g^2 = \hat{\mathbf{w}}'\hat{\mathbf{w}}$
⠀⠀Estimate the per-SNP heritability enrichment for each annotation
⠀⠀Sample residual variance σ_ϵ^2 from its full conditional distribution for each block
**end**
Scale back the posterior mean SNP joint effects to per-allele scale by $\hat{\beta}_j^* = s_j \hat{\beta}_j$

9. Calibrating parameters for the low-rank model

We used simulation to calibrate our low-rank model which requires a specification of two parameters: the minimum width (cM) of a LD block after merging contiguous small quasi-independent LD blocks[1], and the minimum proportion (ρ) of variance in the LD matrix explained by the selected PCs in each LD block. We simulated GWAS data using 1,154,522 SNPs on 328,501 individuals of European ancestry in the UK Biobank[7] (UKB) and calculated the prediction accuracy in a hold-out sample of 14,000 individuals. The results showed that the prediction accuracy increased slightly with the LD block width, reaching to a plateau at 4cM (**Supplementary Fig. 2**). Consequently, we used a minimum block width of 4cM in the subsequent analysis. The other parameter ρ had a greater impact on the prediction accuracy, for which the optimal value would, in principle, depend on the data used for GWAS and LD reference. To determine the best ρ value automatically, we employed a pseudo-validation approach based on the observed GWAS summary statistics and the result of eigen-decomposition on the LD matrix as mentioned below (**Supplementary Note 10**).

10. Tuning for the optimal eigenvalue cut-off

Our low-rank model requires a cut-off value for the minimal proportion (ρ) of variance explained by the eigenvalues in each LD block. The optimal choice of ρ was determined by a summary-data-based validation approach, following the strategy in Zhang et al[8]. First, we constructed a set of summary statistics for training and a set for validation, conditional on the observed GWAS summary statistics and the LD reference data. Second, we carried out multiple short runs (150 iterations) of SBayesRC analysis (without annotations) to estimate the SNP joint effects using the training summary statistics and different ρ values ($\rho = [0.9, 0.95, 0.99, 0.995]'$ by default). Third, we computed the prediction accuracy using the validation summary statistics. Finally, we used the ρ that gave the highest prediction accuracy in the formal analysis. In contrast to Zhang et al, where the strategy of summary-data-based validation was used to tune the model parameters (e.g., $\boldsymbol{\pi}$), we only used this strategy to find the optimal ρ value and still estimate all model parameters from the data. In addition, in Zhang et al, individual-level genotype data from the reference are required, whereas our approach, as described below, only requires the LD correlation matrix without a need to access to the genotype data.

Construction of pseudo summary statistics for training and validation. The observed GWAS summary statistics can be partitioned into training and validation sets by recognising (Zhao et al)

$$X'y = [X_t' \quad X_v'] \begin{bmatrix} y_t \\ y_v \end{bmatrix} = X_t' y_t + X_v' y_v$$

$$\frac{X'y}{n} = \frac{X_t' y_t}{n} + \frac{X_v' y_v}{n}$$

$$\frac{X'y}{n} = \frac{X_t' y_t}{n_t}\frac{n_t}{n} + \frac{X_v' y_v}{n_v}\frac{n_v}{n}$$

$$b = b_t \frac{n_t}{n} + b_v \frac{n_v}{n}$$

assuming standardised genotypes, where the subscript t and v denote the training and validation data sets. Under the assumption of normality and unit phenotypic variance, we have

$$b \sim N(\mu, \frac{1}{n} R)$$

$$b_t \sim N(\mu, \frac{1}{n_t} R)$$

where μ is the true SNP marginal effects. The covariance between b and b_t is

$$\begin{aligned} Cov(b, b_t) &= Cov\left(\frac{X'y}{n}, \frac{X_t' y_t}{n_t}\right) \\ &= \frac{1}{n} Cov(X_t' y_t + X_v' y_v, X_t' y_t) \frac{1}{n_t} \\ &= \frac{1}{n} Var(X_t' y_t) \frac{1}{n_t} \\ &= \frac{n_t}{n} Var\left(\frac{X_t' y_t}{n_t}\right) \\ &= \frac{n_t}{n} Var(b_t) \\ &= \frac{1}{n} R \end{aligned}$$

According to the property of bivariate normal distribution, the conditional expectation and variance of $b_t|b$ are

$$E[b_t|b] = \mu + \Sigma_{b_t,b} \Sigma_b^{-1} (b - \mu) = b$$

$$Var[b_t|b] = \Sigma_{b_t} - \Sigma_{b_t,b} \Sigma_b^{-1} \Sigma_{b_t,b} = \left(\frac{1}{n_t} - \frac{1}{n}\right) R$$

Therefore, the conditional distribution of $b_t|b$ is

$$b_t|b \sim N\left(b, \left(\frac{1}{n_t} - \frac{1}{n}\right) R\right)$$

To obtain samples from this multivariate normal distribution efficiently, we make use of the eigen-decomposition results for each LD block. That is,

$$R = U\Lambda U'$$

where U and Λ are matrices of eigenvectors and eigenvalues with the maximum cut-off of variance explain ($\rho = 0.995$ by default). Let ξ is a vector of standard normal random numbers with the size equal to the number of kept eigenvalues, i.e.,

$$\xi_j \sim N(0,1)$$

Then,

$$b_t|b = b + \sqrt{\frac{1}{n_t} - \frac{1}{n}} U\Lambda^{\frac{1}{2}}\xi$$

The validation data set therefore is

$$b_v|b = b\frac{n}{n_v} - b_t\frac{n_t}{n_v}$$

The marginal standard error for b_t and b_v is simply $\sqrt{1/n_t}$ and $\sqrt{1/n_v}$, respectively.

Calculation of prediction accuracy based on summary statistics. We compute the prediction accuracy as the correlation of phenotypes and PRS in the validation sample, i.e.,

$$R = \frac{Cov(y_v, X_v\beta)}{\sqrt{Var(y_v)}\sqrt{Var(X_v\beta)}}$$

As shown in Zhao et al[9] and Zhang et al[10], the covariance term can be written as a function of validation summary statistics,

$$Cov(y_v, X_v\beta) = \frac{\beta' X_v{}' y_v}{n_v} = \beta' b_v$$

Besides,

$$Var(X_v\beta) = \frac{\beta' X_v{}' X_v\beta}{n_v} = \beta' R\beta \approx mVar(\beta)$$

Putting all together, the prediction accuracy of pseudo-validation can be calculated as

$$R = \frac{\beta' b_v}{\sqrt{mVar(\beta)}}$$

In SBayesRC, we start by running multiple chains with different ρ value of 0.995, 0.99, 0.95, and 0.9, for 150 tunning iterations (posterior mean of β calculated from the last 50 iterations).

We calculate the pseudo-validation R in each chain. If R>0 and |R / R from 0.995| is larger than 1.25, SBayesRC will switch the ρ to the new value from the default of 0.995. If the largest R is from ρ = 0.9, then we will prompt the user to further expand the grid to 0.8, 0.7, 0.6 or check the QC of the summary data.

11. Violation of model assumptions

There are at least two important assumptions implied in the general form of summary-data-based models[6]. One assumption is that the LD correlation matrix calculated from the reference sample is consistent with that from the GWAS sample, which is violated when the LD reference has a too small sample size (i.e., large sampling variation in LD) or is genetically different from the GWAS sample. Another assumption is that the summary statistics are derived from the same set of individuals for all SNPs, which may not hold when the summary statistics are obtained from a meta-analyses where different SNP genotyping panels, imputation references or quality control (QC) procedures are used in different cohorts. Failure to satisfy these assumptions can result in severe model misspecifications. In SBayesRC (or SBayesRC without annotations), we aim to account for the heterogeneity in both LD and per-SNP sample size by removing those principal components with the smallest eigenvalues in the LD matrix and estimating the residual variance from the data (**Methods**). We performed genome-wide simulations based on the imputed SNP data in the UKB to assess the robustness of our method to model misspecifications, in comparison of state-of-the-art methods including LDpred2[5] and SBayesR.

12. Sensitivity analysis in simulation for SBayesRC

For sensitivity analysis, we conducted additional runs of SBayesRC using different mixture distribution scaling factors or the number of mixture components, which deviated from the true values used in the simulation. We observed only negligible changes in the prediction accuracy, indicating that SBayesRC is highly robust to these variations in model specifications (**Supplementary Fig. 9** and **10**). Moreover, to assess the performance of SBayesRC under an alternative data-generative model, we simulated data where the SNP effect variance was modelled as a weighted sum of components based on functional annotations, akin to a model of S-LDSC[11] or MegaPRS[8]. Even in this scenario, SBayesRC demonstrated superior performance (**Supplementary Note 13** and **Supplementary Fig. 10**).

13. Simulation under the S-LDSC/MegaPRS model

We have performed additional simulations where the variance of effect sizes is additively linked to the functional annotations (similar to the LDAK-BayesR-SS or S-LDSC model). The data generative model is:

$$\beta_j \sim \pi_1\delta_0 + \pi_2 N\left(0, s\sigma_j^2/100\right) + \pi_3 N\left(0, s\sigma_j^2/10\right) + \pi_4 N\left(0, s\sigma_j^2\right)$$

Where $s = 1/(\frac{\pi_2}{100} + \frac{\pi_3}{10} + \pi_4)$, $\sigma_j^2 = \sum_c a_{jc}\tau_c^2$ with a_{jc} being the observed annotation per SNP and τ_c^2 being the annotation heritability enrichment parameter from height, and $\pi_1, \pi_2, \pi_3, \pi_4$ are set to be 0.998, 1.89×10^{-3}, 9.26×10^{-6}, and 1.97×10^{-9}, respectively. The trait heritability was set to be 0.1 or 0.5. The pattern of prediction accuracy from using 1M or 7M SNPs and with or without annotations in SBayesRC is similar between the two data generative models (SBayesRC model or S-LDSC model for simulation). In both cases, SBayesRC with 7M SNPs and annotations gave the highest prediction accuracy. We noted that overall the prediction accuracy using the S-LDSC model in the simulation was higher than that using the SBayesRC model in the simulation. This may be because, under the S-LDSC model, some causal variants can have a large causal effect, leading to a large discovery power from GWAS, and therefore higher prediction accuracy. In this case, the added value from the annotations became trivial.

14. Difference between the LDSC/MegaPRS model and SBayesRC model

S-LDSC[12] assumes that each SNP effect has a univariate normal distribution and is not from a mixture model. In this case, it is reasonable to model the SNP effect variance as a weighted sum of components with respect to functional annotations, i.e., $\beta_j \sim N\left(0, \sigma_j^2\right)$, with $\sigma_j^2 = \sum_c a_{jc}\tau_c^2$ where a_{jc} is the value of annotation c at SNP j and τ_c^2 is the heritability enrichment in annotation c. MegaPRS[10] is a tool comprising multiple summary-data-based prediction methods, including an infinitesimal model (LDAK-Ridge-SS), a two-normal mixture model (LDAK-Bolt-SS), and a multi-component mixture model (LDAK-BayesR-SS). LDAK-Ridge-SS can be regarded as an equivalent model as S-LDSC. However, in LDAK-BayesR-SS, $\beta_j \sim \pi_1\delta_0 + \pi_2 N\left(0, \sigma_j^2/100\right) + \pi_3 N\left(0, \sigma_j^2/10\right) + \pi_4 N\left(0, \sigma_j^2\right)$, the biological interpretation is not straightforward. For example, π_2 cannot be interpreted as the proportion of SNPs in the small effect size category because whether the effect size is small or large depends on σ_j^2 which is annotation dependent. This may potentially affect the identifiability of the mixture membership if annotation-specific heritability enrichment parameters τ_c^2 are

estimated jointly with the SNP effects β_j (instead, a two-step estimation approach is used in the MegaPRS paper, where τ_c^2 are estimated by BLD-LDAK model first and then treated as unknown quantities in LDAK-BayesR-SS).

To improve the model interpretability and potentially identifiability and robustness, we assume that

$$\beta_j \sim \pi_{j1}\delta_0 + \pi_{j2}N\left(0, \sigma_g^2/10000\right) + \pi_{j3}N\left(0, \sigma_g^2/1000\right) + \pi_{j4}N\left(0, \sigma_g^2/100\right)$$

where the mixture distributions are independent of annotations, which represent zero, small, medium and large effect sizes that, in order, explains 0, 0.01%, 0.1% and 1% of the total genetic variance (σ_g^2). The mixture distribution membership probabilities, $\pi_{j1}, \pi_{j2}, \pi_{j3}, \pi_{j4}$, are dependent of annotations such that the interpretation for the annotation effects are straightforward, that is, the higher value of the annotation would increase or decrease the probability of the SNP with an effect belong to a given effect size distribution. Since the model is clearly defined, there is no identifiability problem when estimating SNP effects and annotation effects altogether, which we believe is favourable compared to the two-stage estimation approach.

15. Comparison between SBayesRC and MegaPRS

We compared the performance of SBayesRC and MegaPRS (LDAK-BayesR-SS) model using simulations and real data. First, we simulated data based on the SBayesRC model and analysed using the SBayesRC or MegaPRS methods. Second, we simulated data based on the MegaPRS model (or equivalently, the S-LDSC model, when no extra LD weights are modelled) and analysed using the SBayesRC or MegaPRS methods. The details of simulation are described above. We found that SBayesRC gave a higher prediction accuracy than MegaPRS regardless of which data generative model was used in the simulation (**Supplementary Fig. 11**). Third, we ran MegaPRS in the 10-fold cross-validation analysis in the UKB of European ancestry, and found that, although outperformed LDpred2 and LDpred-funt, the prediction accuracy was lower than that from SBayesRC (**Fig. 3a**), consistent with the simulation results. Fourth, we applied MegaPRS in cross-biobank prediction analyses, including training in FinnGen and validation in UKB, and training in the data set from published meta-analysis and validation in Lifelines, across quantitative traits and diseases. Our results showed that SBayesRC had a better predictive performance than MegaPRS, measured by the prediction accuracy relative to LDpred2 (we did not compute the prediction

accuracy relative to the standard SBayesR because of the convergence issue in the standard SBayesR for some of the traits) (**Fig. 3b**). In particular, SBayesRC had a significant advantage over MegaPRS when using an external LD reference independent of the GWAS sample (**Extended Data Fig. 7**). Additionally, the advantage of SBayesRC over MegaPRS is observed with different training sample sizes for height and BMI (**Fig. 3c**). Moreover, our investigation in this study identified a significant interaction between SNP density and annotation information for improving polygenic prediction. We observed this beneficial interactive effect in both within-EUR and cross-ancestry prediction. We note that this is an important result that has not been concluded by the MegaPRS paper, which mainly focused on ~600K directly genotyped SNPs. Last but not least, although MegaPRS is capable of analysing 7M SNPs with annotations, it requires almost 3x larger memory than our method (**Table 1**).

The superiority of SBayesRC may result from three reasons. First, modelling annotations as a function of $\boldsymbol{\pi}$ may be a better way of incorporating annotation data, as explained above. Second, in SBayesRC, all parameters are estimated coherently in one model, whereas MegaPRS takes heritability enrichment estimates and different sets of $\boldsymbol{\pi}$ values as input and do not consider the estimation variance for these parameters. Third, SBayesRC simultaneously fits all SNPs in the model, but MegaPRS only fits a subset of SNPs that are enriched in per-SNP heritability comparing to a random SNP in the genome. SNPs with depletion in heritability enrichment may still have a nonzero, albeit small, effect on the trait, and completely ignoring them may adversely affect prediction accuracy. Last, SBayesRC better accounted for the sampling variation in LD between the reference and GWAS samples through the low-rank approximation, which is likely to give higher prediction accuracy when using an external LD reference that is independent of the GWAS sample.

16. Other factors affecting accuracy of prediction leveraging functional annotations

Here we investigate other factors, besides SNP density, that affect accuracy of prediction leveraging functional annotations, including SNP-based heritability, GWAS sample size, properties of minor allele frequency (MAF) and LD, the number of annotations, and the strategy of analysis.

Stratifying traits based on the SNP-based heritability estimates from SBayesR found that traits with lower SNP-based heritability tended to benefit more from exploiting annotation data on 7M SNPs (regression slope = -15.7, P=0.031; **Fig. 6a**). When down sampling the UKB data for GWAS in height and BMI, the relative prediction accuracy using 7M SNPs and annotations was higher with a smaller GWAS sample size, while using more SNPs alone required a larger sample size to achieve a higher improvement in prediction accuracy (**Fig. 6b**). This is expected because including annotations in the model adds more information, whereas including more SNPs in the model alone increases the number of parameters to estimate, consuming more degrees of freedom. Given that most traits have a polygenic architecture, low SNP-based heritability or small sample size means low power in GWAS. Thus, these results suggest that traits with limited GWAS power would benefit more from leveraging annotation data for prediction.

To investigate whether functional annotations provide additional information compared to LD and MAF properties, the relative prediction accuracy for height and BMI was evaluated by fitting various combinations of annotations. We found that the relative prediction accuracy increased with fitting 13 MAF related annotations and had no significant change when adding 4 LD bins, but further increased with additional functional annotations (**Fig. 6c**). Moreover, when conditional on MAF and LD bins, the improvement was larger with 96 functional annotations compared to 21 core annotations[13]. These results indicate that functional annotations are more informative than LD and MAF annotations, and using a comprehensive set of functional annotations is superior to using only a few key functional categories.

Lastly, we compared alternative strategies of analysing all common SNPs with annotations. Simultaneous fitting of 7M SNPs in the model is computationally impractical in most MCMC-based methods. One strategy is to perform a stepwise analysis[10,14,15], described as below. Step 1, estimation of the impact of functional annotations on SNP effects, which is often quantified by the estimated annotation-specific enrichment in per-SNP heritability using S-LDSC[12]. Step 2, prioritisation of all SNPs based on their functional annotations and the results from step 1. The final analysis uses a subset of the SNPs that rank from the top in Step 2. To compare our method to such a stepwise strategy, we selected the top 1M SNPs from 7M SNP set based on the results from S-LDSC[12], and performed the prediction analysis in height and BMI. For SBayesRC, in which annotations were not assigned, the functionally prioritised 1M SNP set exhibited higher prediction accuracy than the 7M SNPs. However, for

SBayesRC, in which annotations were assigned, the functionally prioritised 1M SNP set was slightly better than the same set of SNPs without re-estimation the annotation effects but was inferior to the 7M SNP set (**Fig. 6d**). These results suggest that the unified analysis using all 7M SNPs in the model is better than the stepwise analysis in refining the information from annotation data.

17. Summary data imputation

We implement the imputation method for summary data from ImpG[16] to avoid the heavy re-calculation of the eigen decomposition for the LD matrix if some SNPs are missing from the LD panel. The imputation is based on the Z score, correlation information among missing SNPs and the typed SNPs. The Z score for the missing SNPs can be obtained from

$$\mathbf{z}_i = \mathbf{R}_{it}\mathbf{R}_{tt}^{-1}\mathbf{Z}_\mathbf{t}$$

Where $\mathbf{z}_i$ is the imputed Z score for missing SNPs in the GWAS summary data, $\mathbf{R}_{it}$ is the LD correlation matrix among the missing SNPs and typed SNPs, $\mathbf{R}_{tt}$ is the LD correlation matrix among the typed SNPs.

We converted the $\mathbf{z}_i$ for missing SNPs to marginal effects at 0/1/2 scale ($\mathbf{b}^*$) and standard error (σ_i) by

$$\sigma_i = \frac{\sigma_y}{\sqrt{2f_i(1-f_i)(N_i + z_i^2)}}$$

$$b^* = z_i\sigma_i$$

Where N_i is the per-SNP sample size (replaced by median per-SNP sample size of known SNPs instead), f_i is the allele frequency from reference genotype, σ_y is the phenotypic standard derivation (square root of phenotypic variance). The phenotypic variance can be estimated by taking the median value of $2f_i(1-f_i)[N_i\sigma_i^2 + (b_i^*)^2]$ across SNPs, here f_i is the allele frequency in the GWAS sample (ref[3,4]).

18. Running settings for the cross-validation in the UKB

SBayesR[17] and LDpred2[5]. Only 1M HapMap3 common SNPs were used due to high computational burden. Chromosome-wise banded LD matrices with a window size of 3cM per SNP were calculated from the LD reference sample. As recommended by the authors of LDpred2, LDpred2 was run with the grid of models setting (96 models), and the hold-out sample of 5,991 UKB individuals with genotypes and phenotypes was used to choose the best model for prediction.

C+PT, LDpred-funct[18], MegaPRS[8], and SBayesRC. Both 1M and 7M common SNP sets were used in these methods. For MegaPRS, the LDAK-BayesR-SS model was used for comparison, as recommended in their paper. For C+PT, a hold-out sample of 5,991 UKB individuals was used as the tunning data, same as in LDpred2. LDpred-funct used the validation sample as the tuning data, which may result in overfitting and inflate the prediction accuracy, so caution is advised. The functional annotation for LDpred-funct, MegaPRS, and SBayesRC was from BaselineLD model v2.2[19], which contains 96 annotations, including functional genomic annotations, such as coding, promoter, enhancer, or conserved variants and MAF related annotations (10 MAFbins, MAF_Adj_Predicted_Allele_Age, MAF_Adj_ASMC and MAF_Adj_LLD_AFR).

19. Running settings for trans-ancestry prediction

1) Model with summary statistics from EUR only.

SBayesR family. We ran SBayesR[17] and SBayesRC using the summary statistics of UKB EUR, and then applied the estimated SNP effects directly to the genotypes of individual of SAS, EAS and AFR ancestries in the UKB.

PolyPred-S. We followed the steps in ref[20], where we first used PolyFun + SuSiE for fine-mapping of 7M SNPs with functional annotations from BaseLineLF and LD data from the PolyPred program calculated from the UKB (a step called PolyFun-pred). Then, the mix SNP weights were optimised in 500 tuning samples of the target ancestry using predictors from both Polyfun-pred and SBayesR (trained in EUR). We obtained the PGS from the optimised SNP weights and the genotypes for each target ancestry (SAS, EAS, and AFR) in the UKB.

To ensure robustness and reduce sampling variation in the relative prediction accuracy, we excluded 10 traits with prediction accuracy (R^2) from SBayesR less than 0.05 in the cross-validation in EUR and removed one trait (age at menarche) that failed to converge with PolyPred-S. As a result, we retained 17 traits for the trans-ancestry prediction analysis.

2) Model summary statistics from two population together.

PRS-CSx[21], SBayesRC-multi, and MegaPRS-multi. We evaluated these methods based on their performance in predicting individuals of EAS and AFR ancestries (target ancestries),

using publicly available GWAS summary statistics from BioBank Japan (BBJ)[22] and Population Architecture using Genomics and Epidemiology (PAGE)[23] datasets. We focused on 8 common traits between our selected traits and BBJ, and another 8 common traits with PAGE. PRS-CSx requires 500 cases in the target tunning samples, but there were not enough case/control samples for disease traits in the UKB of other ancestries, so the analysis was performed only for continuous traits (**Supplementary Table 5** and **6**). The 500 hold-out samples from each of the UKB EAS and AFR were used as the tunning sample. We matched the SNPs in the summary data from BBJ, PAGE, and UKB, and removed SNPs with MAF < 0.005 in either population. After QC, 4,906,538 SNPs remained with functional annotations for EAS, of which 1,011,961 SNPs were in the HapMap3 panel, and 6,064,174 SNPs remained for AFR, of which 1,131,955 SNPs were in the HapMap3 panel. We ran PRS-CSx with GWAS summary statistics from UKB EUR and BBJ populations, and LD data from UKB EUR and EAS downloaded from the website (https://github.com/getian107/PRScsx), using the default parameters. Same pipeline was applied to the analysis of UKB EUR and PAGE AFR datasets with LD data from UKB EUR and AFR. After obtaining the SNP weights specific to EUR and the target (EAS or AFR) populations, we calculated the EUR-based and the target-based PGS with genotypes from the UKB target population. Then, we estimated the optimal weights to combine the two sets of PGS using the target tuning samples with phenotypes, as suggested by PRS-CSx. The prediction accuracy was calculated from the optimised PGS and phenotypes in the remaining target validation samples. Following a similar strategy, we extended our method to utilise GWAS data from multiple populations (SBayesRC-multi) and did the same for MegaPRS-multi. Instead of joint modelling, we ran SBayesRC or MegaPRS using summary statistics from UKB EUR and LD from UKB EUR. Then, we ran the methods using summary statistics from BBJ or PAGE with LD from UKB EAS or AFR separately. Finally, the final SNP effects for the target individuals were derived by combining the EUR- and the target-based PGS in the target tuning samples, as described above.

20. Reason for using cross-validation in the UKB analysis

The k-fold cross-validation approach has been widely used in the PRS analysis. We reported the mean prediction R^2 across 10 folds and believe this is more reliable to the result from a single random sample for the training set with the remaining being the validation set, because the mean value is expected to average out potential ascertainment bias in partitioning training

and validation samples. In addition, cross-validation provides a way to compute the standard error of the prediction accuracy for a trait, which is important when comparing differences in prediction performance between models/scenarios. For example, as shown in **Fig. 5** of the main text, the improvement in prediction accuracy due to the use of annotations was significantly higher at 7M imputed SNPs than 1M HapMap3 SNPs for each trait, which can be seen from the standard error obtained from the cross-validation approach.

The reason we used 342K unrelated (genomic relationship cut-off at 0.05) individuals only is that SNPs can capture both direct and indirect genetic effects in the family as well as family-specific environmental effects, which will, if validation samples include relatives of training samples, lead to a higher prediction accuracy than that would be expected in a random sample of the population. Prediction accuracy in an independent random sample remains the most widely accepted criterion to assess the PRS performance. Therefore, we wished to only include the unrelated individuals in the validation set and did not use data from other individuals beyond the 342K unrelated samples of European ancestry.

21. Acknowledgements

Lifelines Cohort Study

The Lifelines Biobank initiative has been made possible by funding from the Dutch Ministry of Health, Welfare and Sport, the Dutch Ministry of Economic Affairs, the University Medical Center Groningen (UMCG the Netherlands), University of Groningen and the Northern Provinces of the Netherlands. The generation and management of GWAS genotype data for the Lifelines Cohort Study is supported by the UMCG Genetics Lifelines Initiative (UGLI). UGLI is partly supported by a Spinoza Grant from NWO, awarded to Cisca Wijmenga. The authors wish to acknowledge the services of the Lifelines Cohort Study, the contributing research centers delivering data to Lifelines, and all the study participants.

UK Biobank

This study has been conducted using UK Biobank resource under Application Number 12514. UK Biobank was established by the Wellcome Trust medical charity, Medical Research Council, Department of Health, Scottish Government and the Northwest Regional Development Agency. It has also had funding from the Welsh Assembly Government, British Heart Foundation and Diabetes UK.

FinnGen Study

The FinnGen study is a large-scale genomics initiative that has analyzed over 500,000 Finnish biobank samples and correlated genetic variation with health data to understand disease mechanisms and predispositions. The project is a collaboration between research organisations and biobanks within Finland and international industry partners. We want to acknowledge the participants and investigators of the FinnGen study.

Supplementary Figures

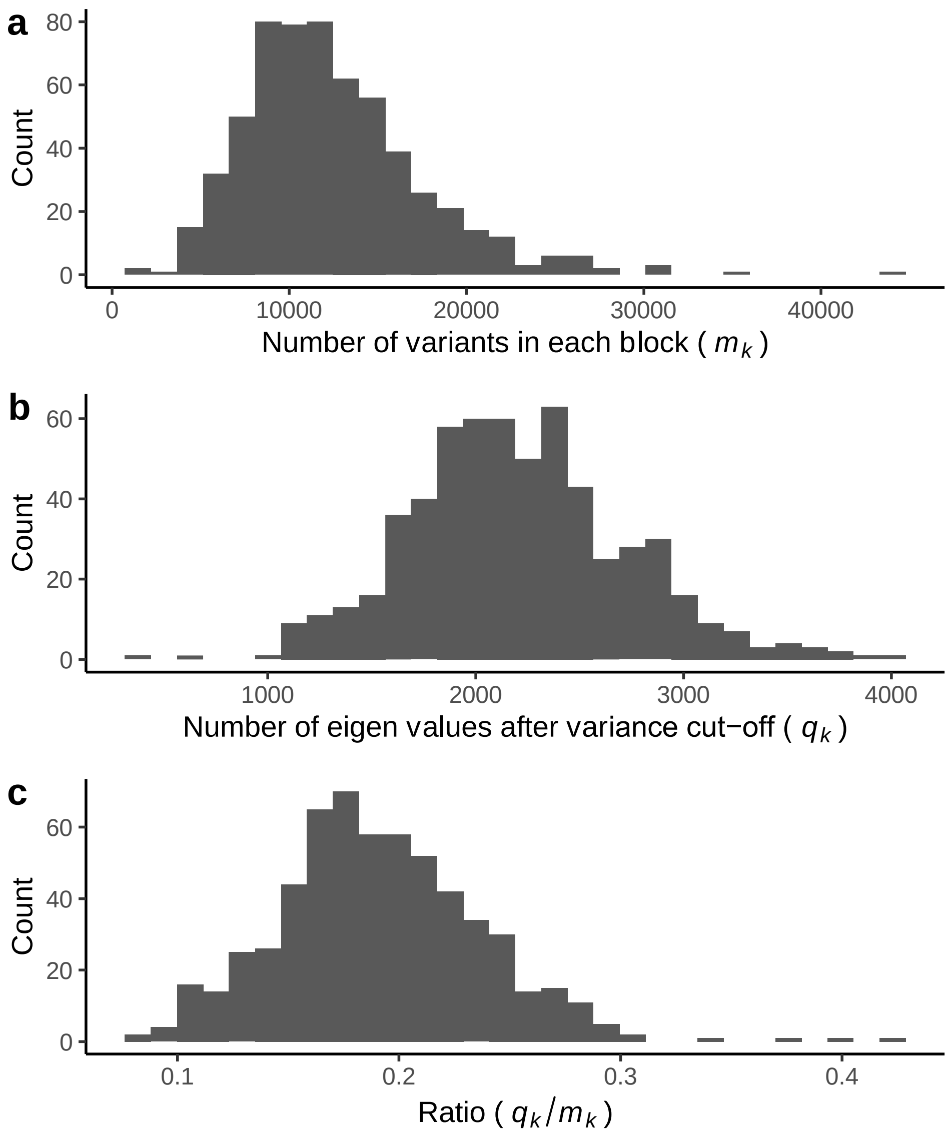

Supplementary Figure 1 The low-rank model leads to a substantial reduction in dimension. a) The distribution of the number of SNPs per block (m_k). b) The distribution of the number of principal components (q_k) that collectively explain at least ρ proportion of LD variance in each block ($\rho = 99.5\%$). c) The distribution of q_k/m_k at $\rho = 99.5\%$.

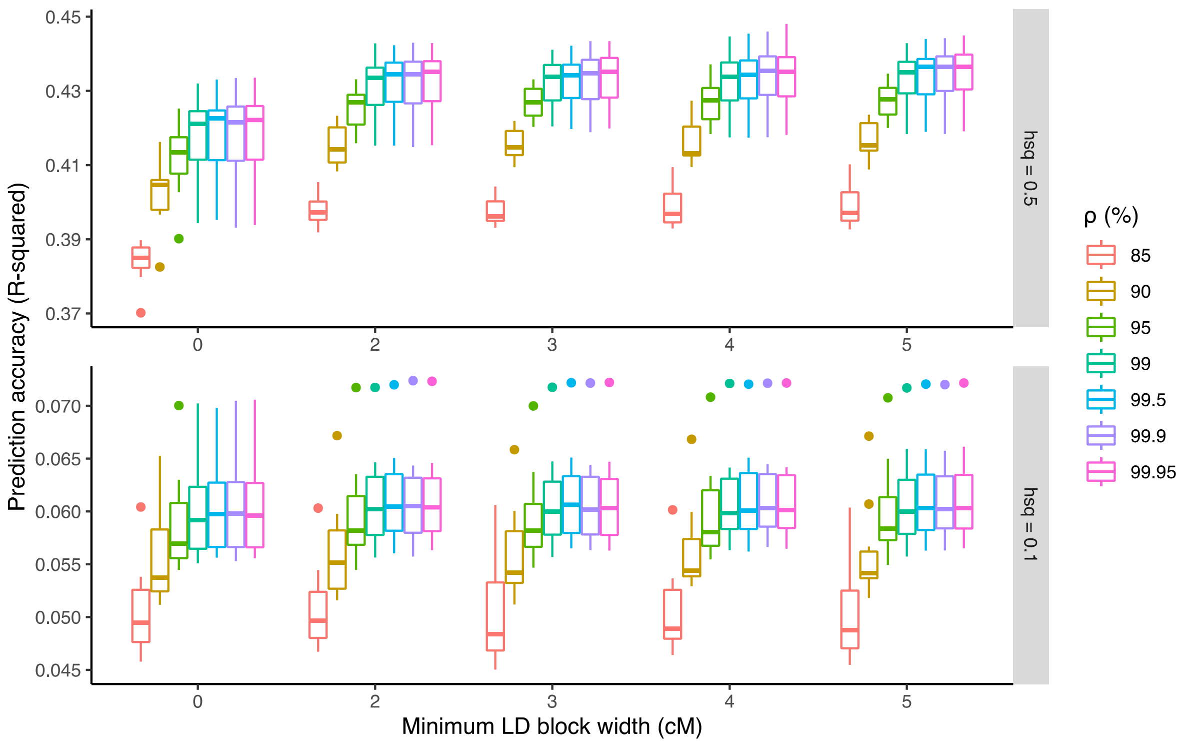

Supplementary Figure 2 Prediction accuracy of SBayesRC using different minimum values of LD block width and different minimum proportions (ρ) of variance in the LD matrix in the simulated data with heritability = 0.1 or 0.5. Minimum LD block width = 0 means using the original quasi-independent LD blocks found in the European population (ref[1]) without merging of small LD blocks. Each box plot shows the spread of data in 10 independent simulations: the line is the middle (median), the box covers the middle half (IQR), the whiskers extend to 1.5 times the IQR, and dots show outliers.

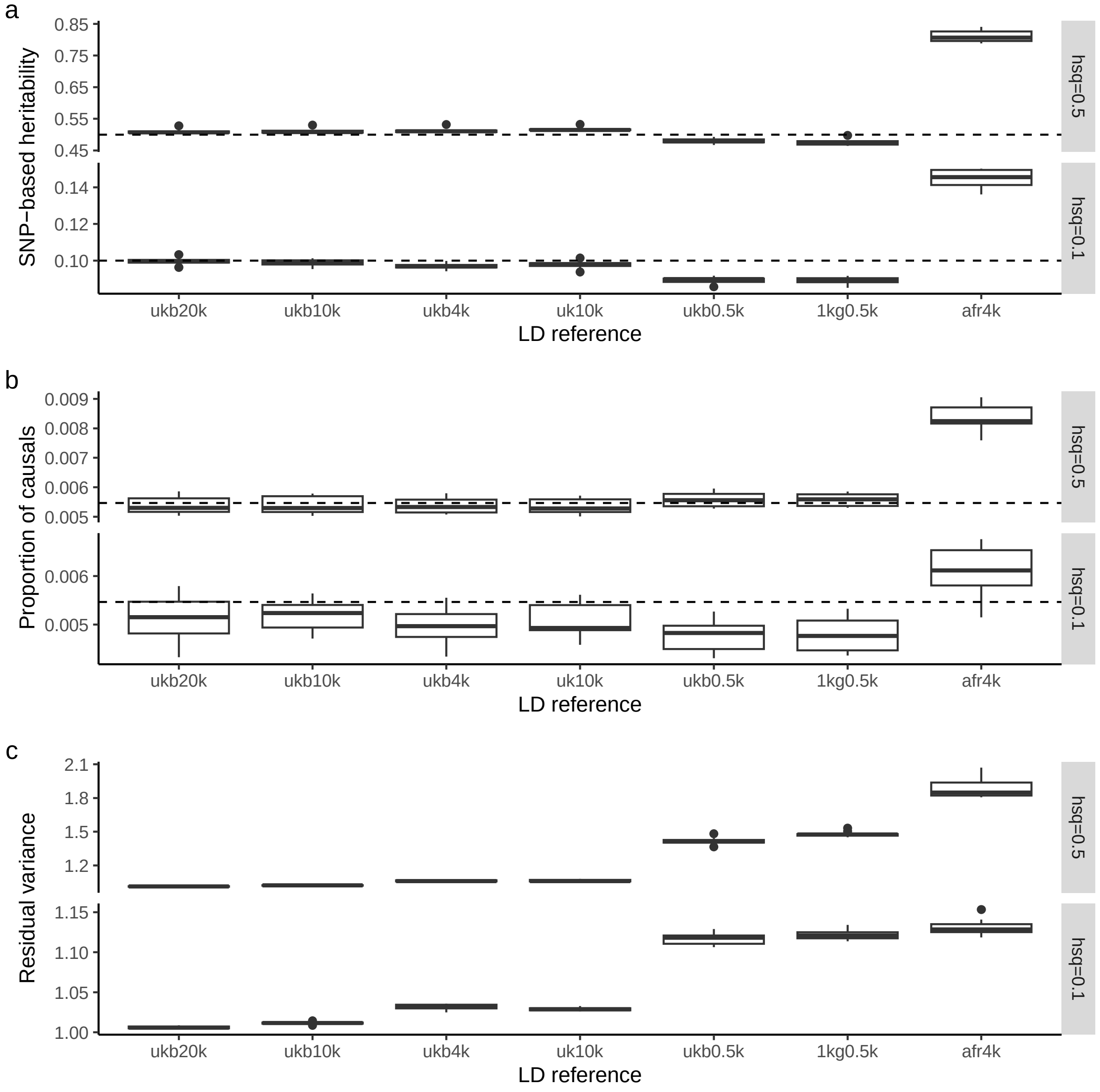

Supplementary Figure 3 Estimation of SNP-based heritability, polygenicity (the proportion of causal variants) and residual variance in SBayesRC without annotation using1M HapMap3 SNPs and different choices of LD reference for a simulated trait with heritability = 0.1 or 0.5. The dashed line in panel a and b indicates the true value in the simulation. The dashed line shows the simulated value. Each box plot shows the spread of data in 10 independent simulations: the line is the middle (median), the box covers the middle half (IQR), the whiskers extend to 1.5 times the IQR, and dots show outliers.

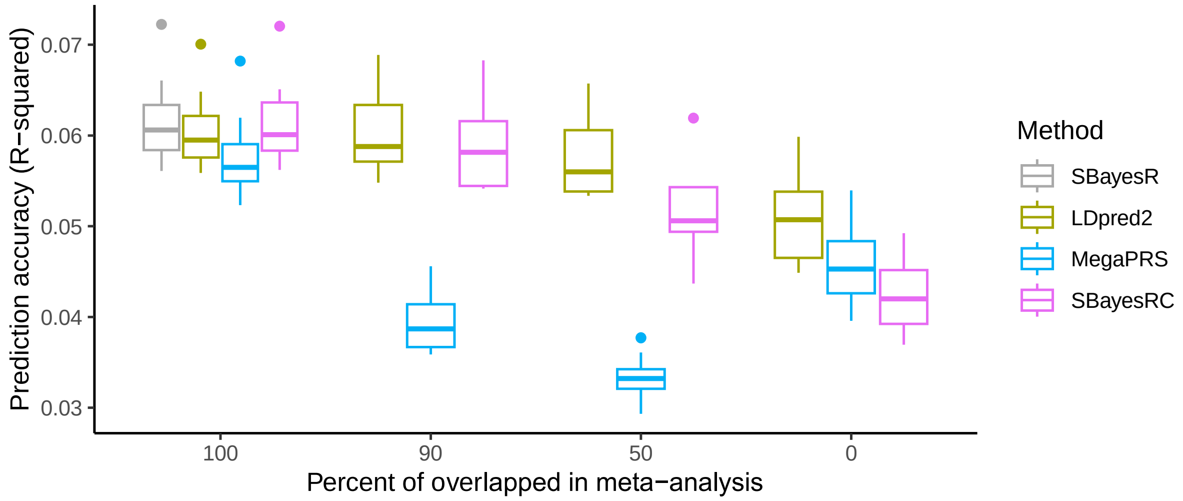

Supplementary Figure 4 Robustness of SBayesRC to unequal per-SNP sample sizes in meta-analysis through simulations with a trait heritability of h^2=0.1. While SBayesR only converged in the case of 100% SNP overlap, SBayesRC was able to reach convergence in all scenarios. When the trait heritability is low (h^2=0.1), methods lacking tuning information (MegaPRS and SBayesRC) exhibited poorer performance, compared to methods incorporating tuning samples (LDpred2-grid). This observation suggests that methods utilizing tuning samples excel in the case of model misspecification when the GWAS power is low. Each box plot shows the spread of data in 10 independent simulations: the line is the middle (median), the box covers the middle half (IQR), the whiskers extend to 1.5 times the IQR, and dots show outliers.

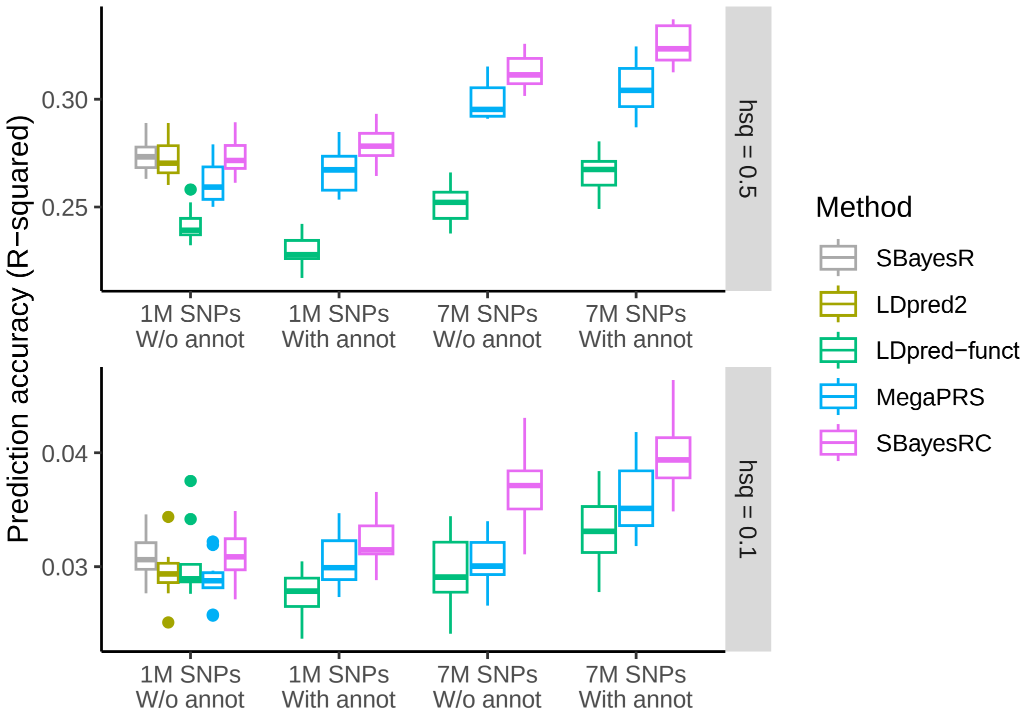

Supplementary Figure 5 Prediction accuracy of phenotypes using PGS derived from different methods for simulated traits (heritability = 0.1 or 0.5). Each box plot shows the spread of data in 10 independent simulations: the line is the middle (median), the box covers the middle half (IQR), the whiskers extend to 1.5 times the IQR, and dots show outliers.

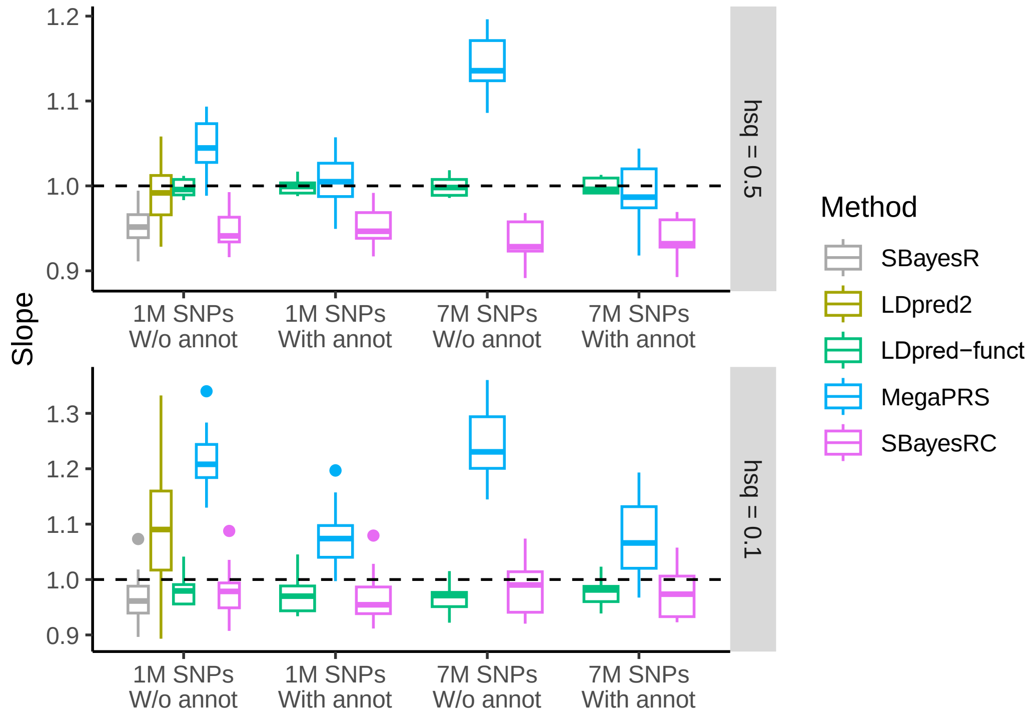

Supplementary Figure 6 Slope of regression (bias) of phenotypes using PGS derived from different methods and SNP panels using the simulated data. The dashed line indicates the slope of 1. Each box plot shows the spread of data in 10 independent simulations: the line is the middle (median), the box covers the middle half (IQR), the whiskers extend to 1.5 times the IQR, and dots show outliers.

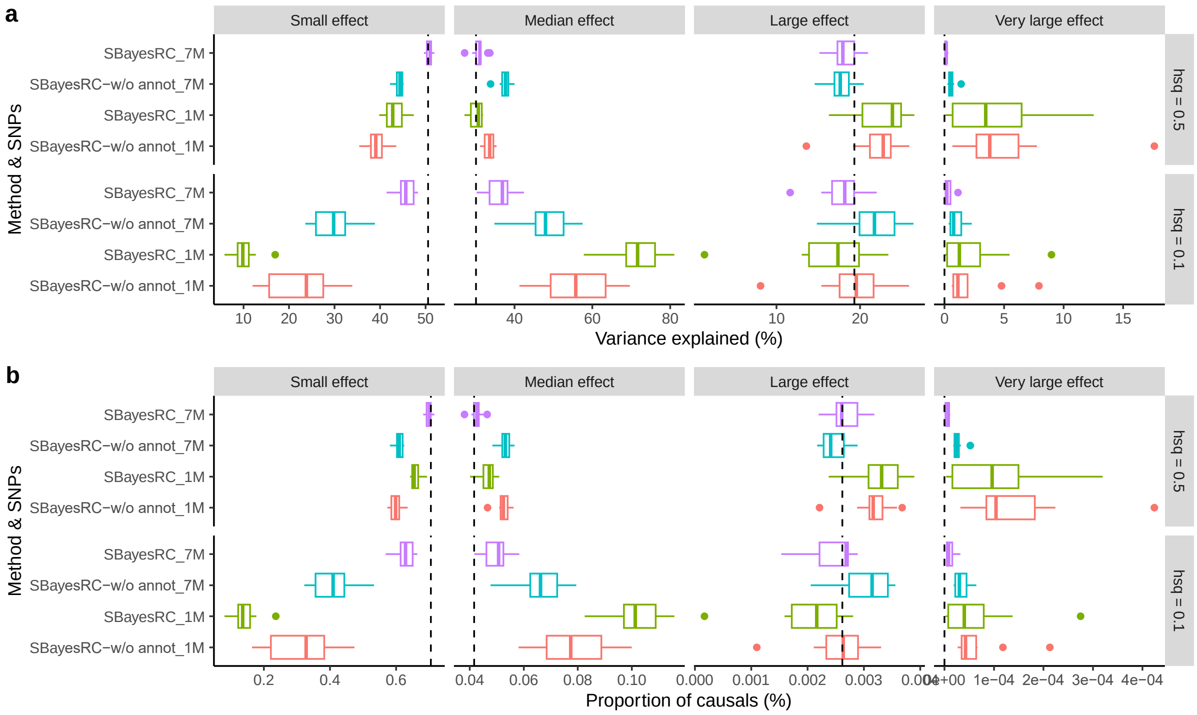

Supplementary Figure 7 Genetic architecture parameter estimation using SBayesRC without annotation or SBayesRC (incorporating annotation data) with different SNP panels for a simulated trait (heritability = 0.1 or 0.5). The dashed line indicates the true value in the simulation. Each box plot shows the spread of data in 10 independent simulations: the line is the middle (median), the box covers the middle half (IQR), the whiskers extend to 1.5 times the IQR, and dots show outliers.

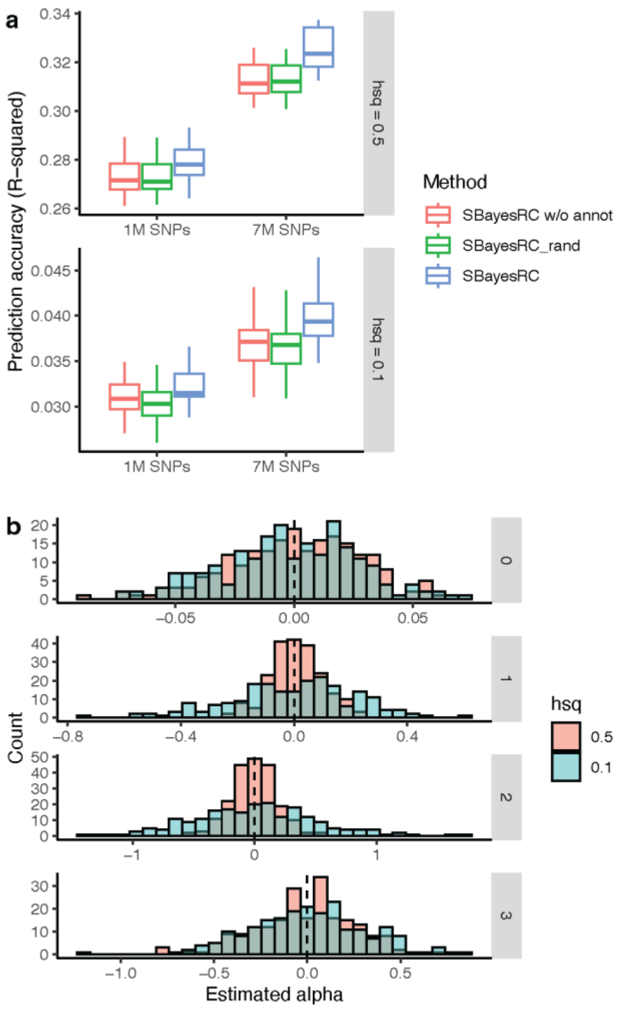

Supplementary Figure 8 The effect of random annotations. a) SBayesRC improved prediction accuracy due to incorporation of annotation data, evidenced by the same analysis using a random annotation as negative control for a simulated trait (heritability = 0.5 or 0.1). SBayesRC_rand is SBayesRC when random numbers sampled from a uniform distribution between 0 and 1 are used as annotation data (a negative control for SBayesRC). Each box plot shows the spread of data in 10 independent simulations: the line is the middle (median), the box covers the middle half (IQR), the whiskers extend to 1.5 times the IQR, and dots show outliers. b) The histogram of estimated annotation effects (alpha) when using random annotations in the simulation (trait heritability h^2=0.5 or 0.1). The dashed line indicated the true value of zero (random annotations have no effect on the mixing probabilities of SNP effect distribution). Rows 0-3 indicated the simulated components of mixture distribution for the SNP effects. As expected, the estimated annotation effects were centred at zero in each component. However, when the power is relatively low (h^2=0.1), the estimated annotation effects were more dispersed, resulting in a slight decrease in prediction accuracy, as shown in panel a.

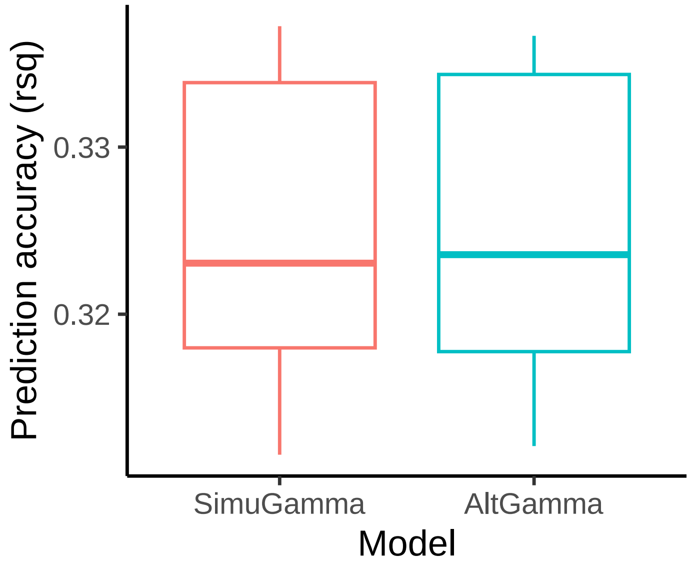

Supplementary Figure 9 Prediction accuracy from SBayesRC using a different set of mixture distribution scaling factors ($\boldsymbol{\gamma}$) than the true values for simulating the data. SimuGamma shows the result with $\boldsymbol{\gamma}$ values identical to the true values in the simulation $\boldsymbol{\gamma}$ = [0, 0.001, 0.01, 0.1, 1]. AltGamma shows the result with $\boldsymbol{\gamma} = [0, 0.001, 0.005, 0.01, 0.02]$. Each box has the result from 10 simulation replicates. Each box plot shows the spread of data in 10 independent simulations: the line is the middle (median), the box covers the middle half (IQR), the whiskers extend to 1.5 times the IQR, and dots show outliers.

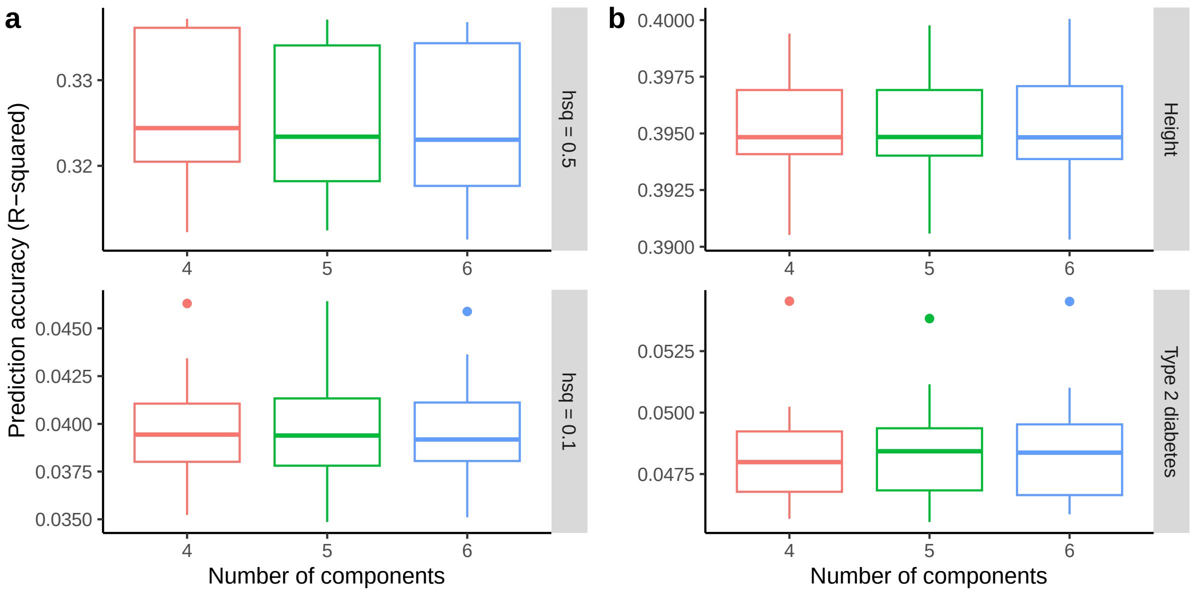

Supplementary Figure 10 Prediction accuracy with 4 (gamma: [0, 0.001, 0.01, 0.1]), 5 (gamma: [0, 0.001, 0.01, 0.1, 1]), and 6 (gamma: [0, 0.001, 0.01, 0.1, 0.5, 1] components in simulation and real traits. a) Simulated traits based on 5 components (heritability 0.5 or 0.1); b) Height and type 2 diabetes in the UKB cross-validation. The difference in prediction accuracy is negligible for both simulated and real traits. Each box plot shows the spread of data in 10 independent simulations: the line is the middle (median), the box covers the middle half (IQR), the whiskers extend to 1.5 times the IQR, and dots show outliers.

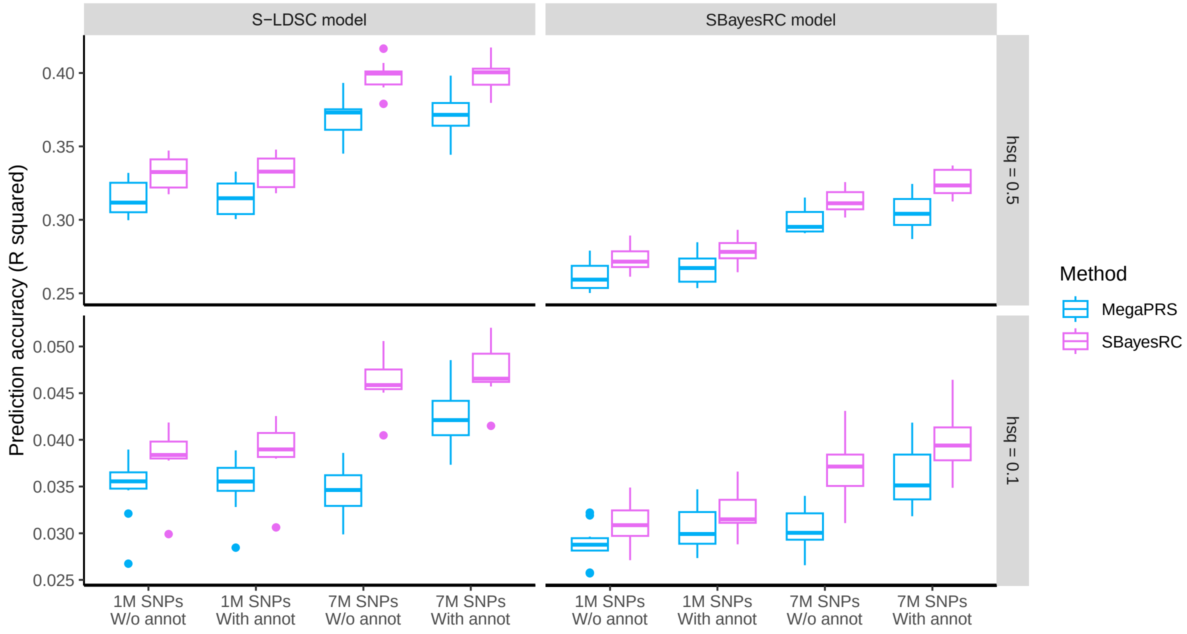

Supplementary Figure 11 Prediction accuracy of SBayesRC in comparison to MegaPRS in the simulations where data were generated using either SBayesRC or S-LDSC model (simulated heritability = 0.5 or 0.1). Each box plot shows the spread of data in 10 independent simulations: the line is the middle (median), the box covers the middle half (IQR), the whiskers extend to 1.5 times the IQR, and dots show outliers. See the **Supplementary Note** for details of simulation based on S-LDSC model.

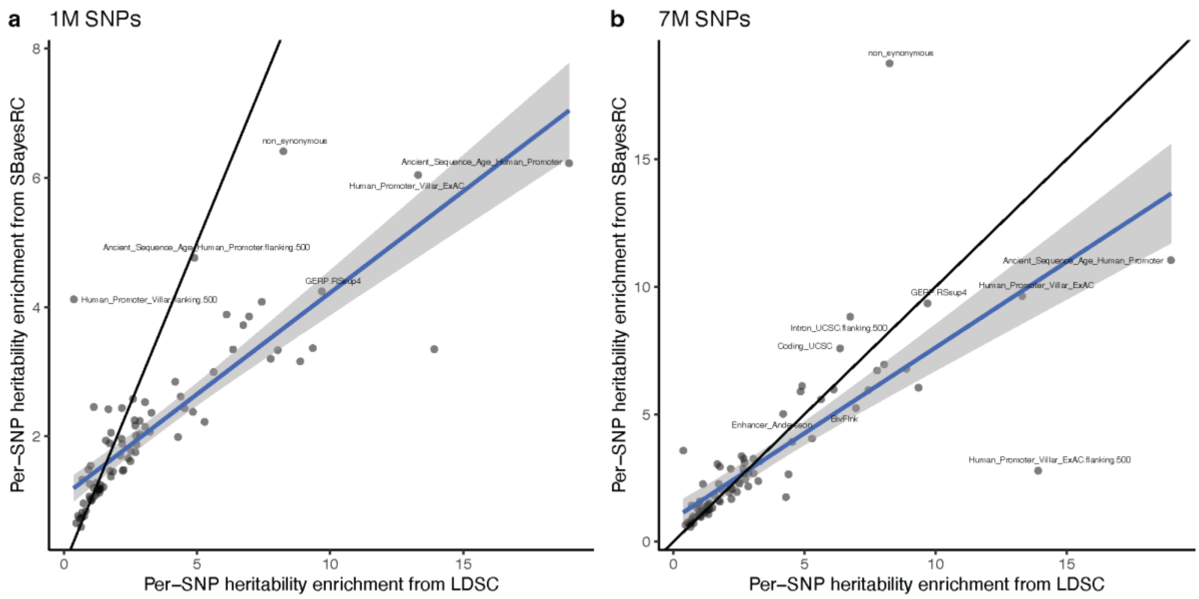

Supplementary Figure 12 Comparison of per-SNP heritability enrichment in functional categories estimated by SBayesRC and S-LDSC using 1M (panel a) or 7M (panel b) SNPs. The result of each functional category was the mean value over 28 independent UKB traits. The black solid line indicates y=x, the blue solid line indicates the regression line of the data points, the shading is the confident interval of the regression line.

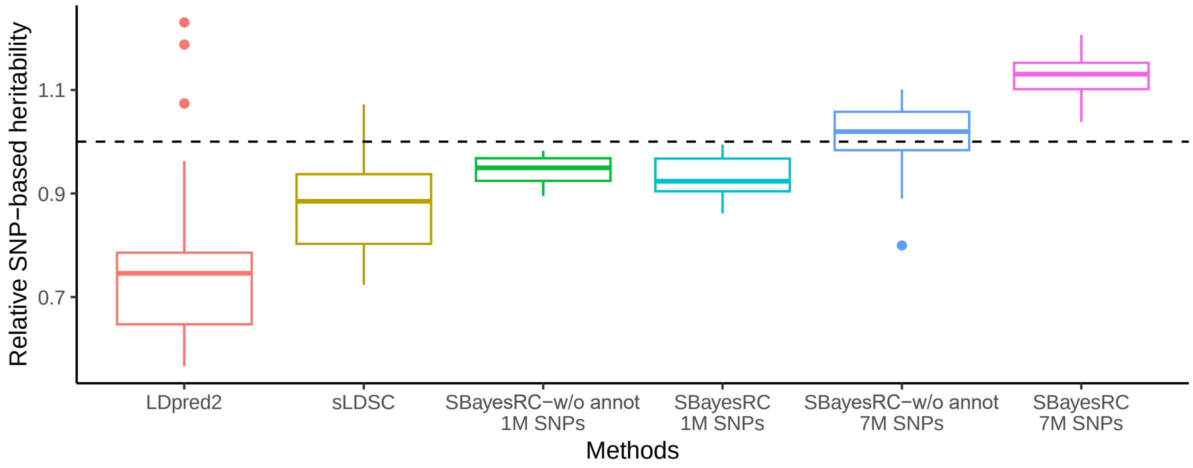

Supplementary Figure 13 Relative SNP-based heritability estimate from different methods (baseline: estimate from SBayesR) for 28 independent traits in UKB unrelated European sample. The dashed line indicates the relative SNP-based heritability equal to 1 (estimation from the method equals to SBayesR). Each box plot shows the spread of data in 28 independent traits: the line is the middle (median), the box covers the middle half (IQR), the whiskers extend to 1.5 times the IQR, and dots show outliers.

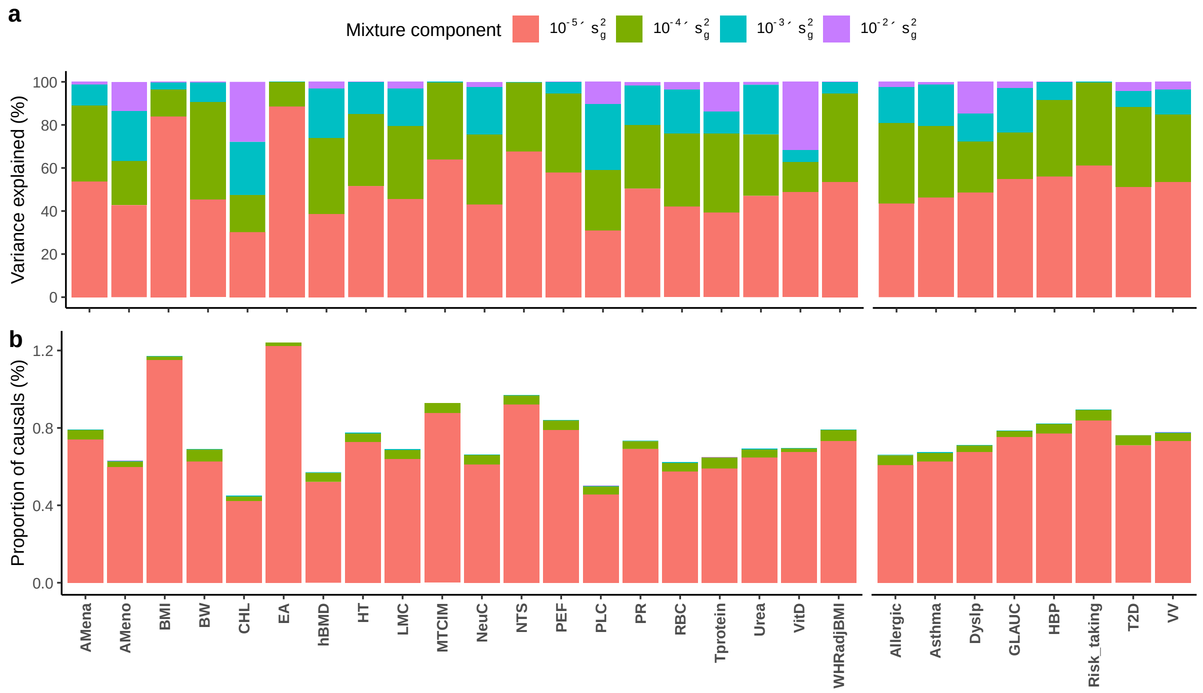

Supplementary Figure 14 Genetic architecture estimates from SBayesRC using 7M common SNPs and annotation data for 28 independent traits in the UKB unrelated European sample. a) The proportion of genetic variance explained in the four non-zero mixture components. b) The proportion of causal variants allocated in the four non-zero mixture components.

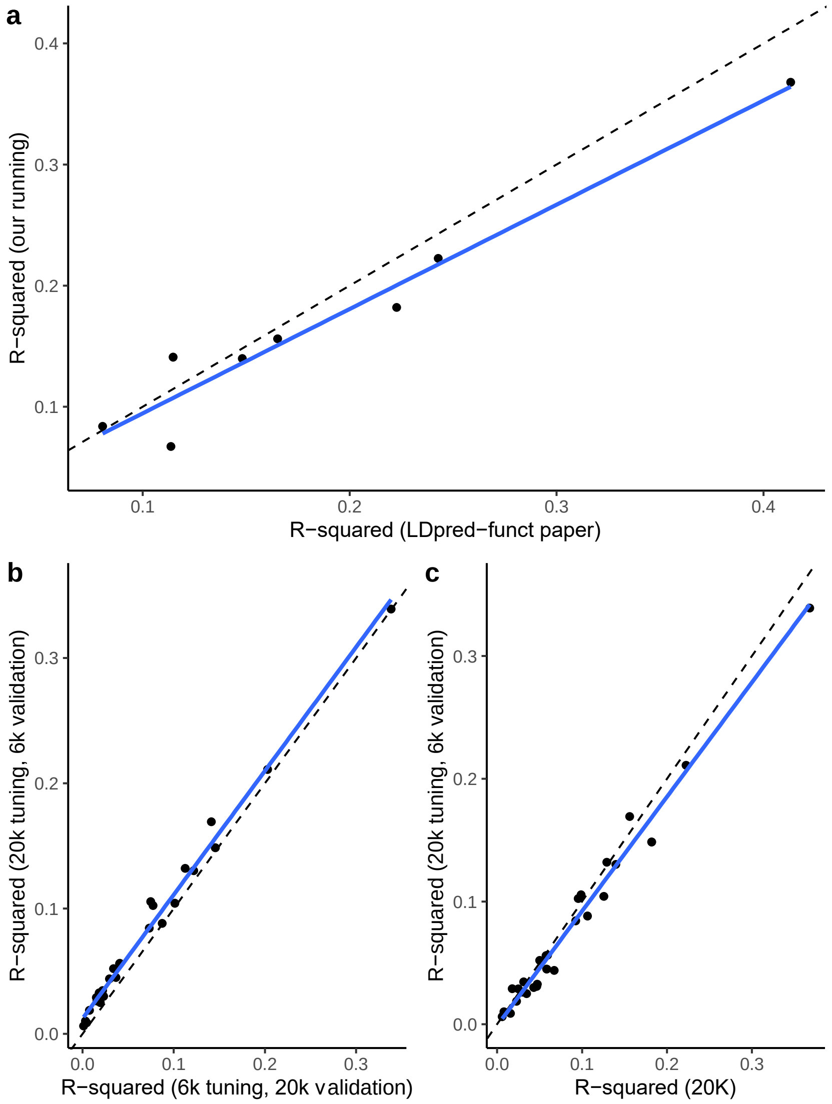

Supplementary Figure 15 Comparison of prediction accuracy using LDpred-funct from this analysis to that reported in the LDpred-funct paper for the same trait. a) Comparison of prediction accuracy from our running (mean N=282,019) and that from the LDpred-funct paper (mean N=390,208). Although the prediction accuracies were highly correlated (r=0.975), we found a somewhat lower prediction accuracy in most traits, likely because of the smaller sample size used in this study. b) We found that a larger tuning sample size of 20K consistently gave better result than that of 6K in LDpred-funct. c) The default setting in LDpred-funct is to use the validation sample as tuning (x-axis), which gave slightly better prediction accuracy than using an independent validation sample but is subject to overfitting. The black dashed line indicates y=x, the blue solid line indicates the regression line of the data points, the shading is the confident interval of the regression line.

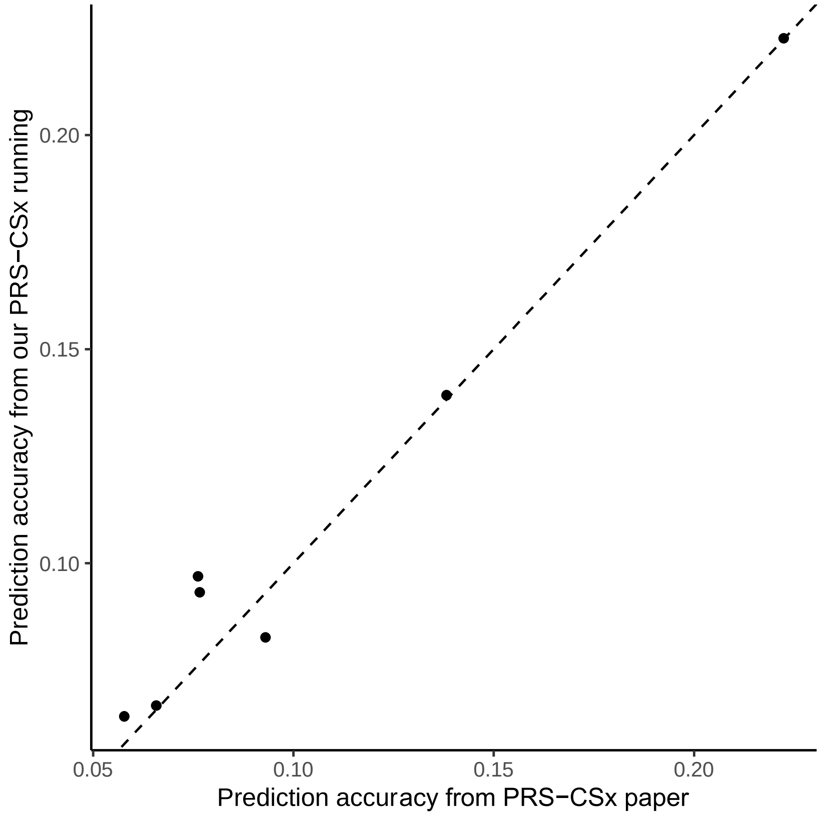

Supplementary Figure 16 Comparison of prediction accuracy (R^2) using PRS-CSx from this analysis to that reported in the PRS-CSx paper for the same traits in EAS population training by summary data from UKB (EUR) and BBJ (EAS). The black dashed line indicates y=x.

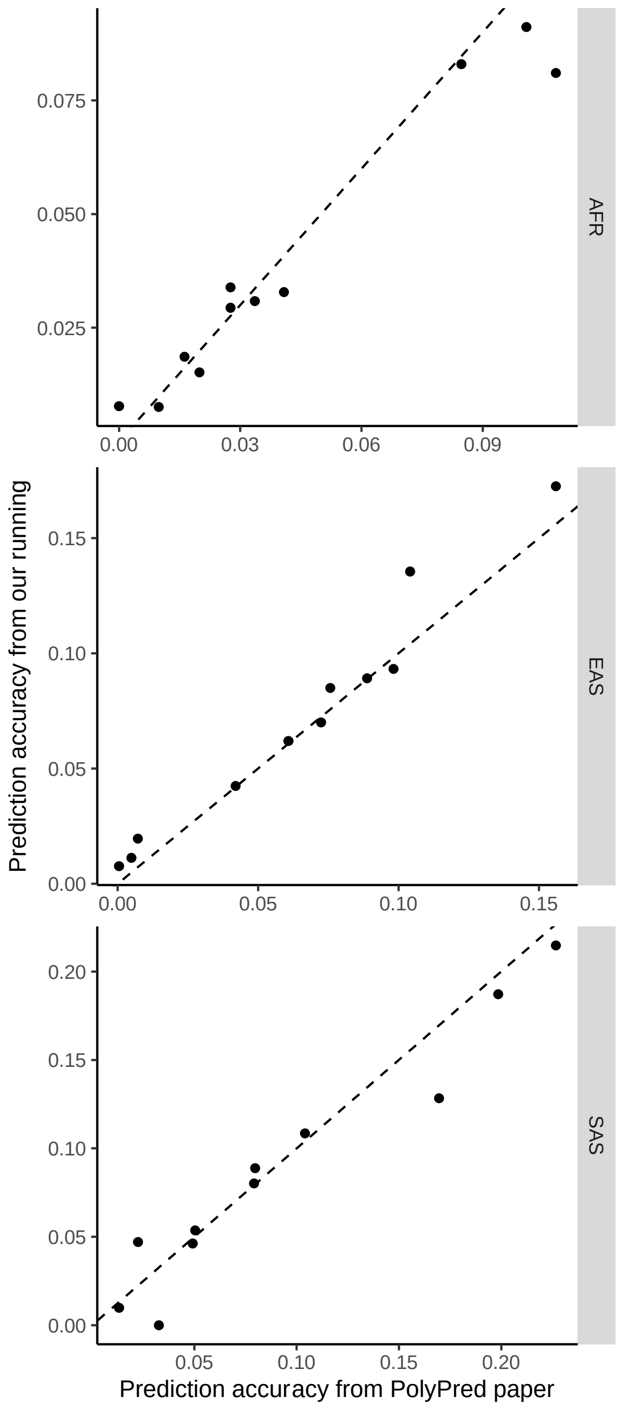

Supplementary Figure 17 Comparison of prediction accuracy (R^2) using PolyPred-S from this analysis to that reported in the PolyPred paper for the same traits across ancestries. The black dashed line indicates y=x. The prediction accuracies were highly correlated (r = 0.976). The differences may come from 1) Different ways to generate the summary statistics (linear regression/ logistic regression vs. linear mixed model); 2) Differences in training SNP panel (7 million vs. 18 million). We have also discussed with the authors to correct potential issues (https://github.com/omerwe/polyfun/issues/80).

Reference

1. Berisa, T. & Pickrell, J.K. Approximately independent linkage disequilibrium blocks in human populations. *Bioinformatics (Oxford, England)* **32**, 283-285 (2016).
2. Albert, J.H. & Chib, S. Bayesian-Analysis of Binary and Polychotomous Response Data. *Journal of the American Statistical Association* **88**, 669-679 (1993).
3. Lloyd-Jones, L.R. *et al.* Improved polygenic prediction by Bayesian multiple regression on summary statistics. *Nature Communications* **10**, 5086 (2019).
4. Yang, J. *et al.* Conditional and joint multiple-SNP analysis of GWAS summary statistics identifies additional variants influencing complex traits. *Nat Genet* **44**, 369-75, S1-3 (2012).
5. Prive, F., Arbel, J. & Vilhjalmsson, B.J. LDpred2: better, faster, stronger. *Bioinformatics* (2020).
6. Zhu, X. & Stephens, M. Bayesian Large-Scale Multiple Regression with Summary Statistics from Genome-Wide Association Studies. *Ann Appl Stat* **11**, 1561-1592 (2017).
7. Bycroft, C. *et al.* The UK Biobank resource with deep phenotyping and genomic data. *Nature* **562**, 203-+ (2018).
8. Zhang, Q., Prive, F., Vilhjalmsson, B. & Speed, D. Improved genetic prediction of complex traits from individual-level data or summary statistics. *Nat Commun* **12**, 4192 (2021).
9. Zhao, Z. *et al.* PUMAS: fine-tuning polygenic risk scores with GWAS summary statistics. *Genome Biol* **22**, 257 (2021).
10. Zhang, Q.Q., Prive, F., Vilhjalmsson, B. & Speed, D. Improved genetic prediction of complex traits from individual-level data or summary statistics. *Nature Communications* **12**(2021).
11. Finucane, H.K. *et al.* Partitioning heritability by functional annotation using genome-wide association summary statistics. *Nat Genet* **47**, 1228-35 (2015).
12. Finucane, H.K. *et al.* Partitioning heritability by functional annotation using genome-wide association summary statistics. *Nature Genetics* **47**, 1228-1235 (2015).
13. Gazal, S., Marquez-Luna, C., Finucane, H.K. & Price, A.L. Reconciling S-LDSC and LDAK functional enrichment estimates. *Nature Genetics* **51**, 1202-1204 (2019).
14. Xiang, R. *et al.* Quantifying the contribution of sequence variants with regulatory and evolutionary significance to 34 bovine complex traits. *Proc Natl Acad Sci U S A* **116**, 19398-19408 (2019).
15. Maarquez-Luna, C. *et al.* Incorporating functional priors improves polygenic prediction accuracy in UK Biobank and 23andMe data sets. *Nature Communications* **12**(2021).
16. Pasaniuc, B. *et al.* Fast and accurate imputation of summary statistics enhances evidence of functional enrichment. *Bioinformatics* **30**, 2906-14 (2014).
17. Lloyd-Jones, L.R. *et al.* Improved polygenic prediction by Bayesian multiple regression on summary statistics. *Nat Commun* **10**, 5086 (2019).
18. Marquez-Luna, C. *et al.* Incorporating functional priors improves polygenic prediction accuracy in UK Biobank and 23andMe data sets. *Nat Commun* **12**, 6052 (2021).
19. Gazal, S. *et al.* Linkage disequilibrium-dependent architecture of human complex traits shows action of negative selection. *Nature Genetics* **49**, 1421-+ (2017).
20. Weissbrod, O. *et al.* Leveraging fine-mapping and multipopulation training data to improve cross-population polygenic risk scores. *Nature Genetics* **54**, 450-+ (2022).

21. Ruan, Y.F. *et al.* Improving polygenic prediction in ancestrally diverse populations. *Nature Genetics* **54**, 573-+ (2022).
22. Sakaue, S. *et al.* A cross-population atlas of genetic associations for 220 human phenotypes. *Nature Genetics* **53**, 1415-+ (2021).
23. Wojcik, G.L. *et al.* Genetic analyses of diverse populations improves discovery for complex traits. *Nature* **570**, 514-+ (2019).
