# Supplementary material for: Leveraging functional genomic annotations and genome coverage to improve polygenic prediction of complex traits within and between ancestries
Source: Nat Genet. 2024 Apr 30;56(5):767–77. doi: 10.1038/s41588-024-01704-y (PMC11096109; doi:10.1038/s41588-024-01704-y)
Supplement: Supplementary file 2 — Reporting Summary [file 41588_2024_1704_MOESM2_ESM.pdf]

nature portfolio

Corresponding author(s): Zhili Zheng, Jian Zeng

Last updated by author(s): Dec 21, 2023

# Reporting Summary

Nature Portfolio wishes to improve the reproducibility of the work that we publish. This form provides structure for consistency and transparency in reporting. For further information on Nature Portfolio policies, see our Editorial Policies and the Editorial Policy Checklist.

## Statistics

For all statistical analyses, confirm that the following items are present in the figure legend, table legend, main text, or Methods section.

| n/a | Confirmed | |
|---|---|---|
| ☐ | ☒ | The exact sample size ($n$) for each experimental group/condition, given as a discrete number and unit of measurement |
| ☒ | ☐ | A statement on whether measurements were taken from distinct samples or whether the same sample was measured repeatedly |
| ☐ | ☒ | The statistical test(s) used AND whether they are one- or two-sided<br>*Only common tests should be described solely by name; describe more complex techniques in the Methods section.* |
| ☐ | ☒ | A description of all covariates tested |
| ☐ | ☒ | A description of any assumptions or corrections, such as tests of normality and adjustment for multiple comparisons |
| ☐ | ☒ | A full description of the statistical parameters including central tendency (e.g. means) or other basic estimates (e.g. regression coefficient) AND variation (e.g. standard deviation) or associated estimates of uncertainty (e.g. confidence intervals) |
| ☐ | ☒ | For null hypothesis testing, the test statistic (e.g. $F$, $t$, $r$) with confidence intervals, effect sizes, degrees of freedom and $P$ value noted<br>*Give P values as exact values whenever suitable.* |
| ☐ | ☒ | For Bayesian analysis, information on the choice of priors and Markov chain Monte Carlo settings |
| ☒ | ☐ | For hierarchical and complex designs, identification of the appropriate level for tests and full reporting of outcomes |
| ☐ | ☒ | Estimates of effect sizes (e.g. Cohen's $d$, Pearson's $r$), indicating how they were calculated |

*Our web collection on statistics for biologists contains articles on many of the points above.*

## Software and code

Policy information about availability of computer code

| | |
|---|---|
| Data collection | No data collection software was used in this study. The data was described in the Methods of the manuscript, which was collected by multiple Biobanks. |
| Data analysis | We used GCTA v1.93.2, PLINK2 v2.00a2.3 and R 4.0.4 for the quality control of individual level data and generate the summary statistics as described in the Methods section. We obtained the SNP weights from summary statistics via SBayesRC-v0.2.0(https://github.com/zhilizheng/SBayesRC), PLINK v1.90b6.21, LDpred2 v1.8.1, GCTB v2.03, PolyPred (Feb 1, 2022), LDpred-funct (Aug 30, 2021), MegaPRS (v5.2) and PRS-CSx (Jun 29, 2022). The polygenic scores were calculated by PLINK2 v2.00a2.3 from the weights. Refer to the links in the main text to all the analysis tools and Methods section for details. |

For manuscripts utilizing custom algorithms or software that are central to the research but not yet described in published literature, software must be made available to editors and reviewers. We strongly encourage code deposition in a community repository (e.g. GitHub). See the Nature Portfolio guidelines for submitting code & software for further information.

## Data

Policy information about availability of data

All manuscripts must include a data availability statement. This statement should provide the following information, where applicable:

- Accession codes, unique identifiers, or web links for publicly available datasets
- A description of any restrictions on data availability
- For clinical datasets or third party data, please ensure that the statement adheres to our policy

We have updated the Data Availability statement in the manuscript:
The UK Biobank data, UK10K and LifeLines are available through formal application to the UK Biobank (http://www.ukbiobank.ac.uk), UK10K (https://www.uk10k.org/data_access.html) and LifeLines (https://www.lifelines.nl/researcher/how-to-apply). The summary data and PGS weights from SBayesRC for the 28 approximately independent UKB traits can be found at https://cnsgenomics.com/software/gctb/#Download. All the other datasets used in this study are available in the public domain. 1000 Genomes: https://www.internationalgenome.org/data/; FinnGen: https://www.finngen.fi/en/access_results (version: R8); BBJ: https://biobankjp.org/en/ ; PAGE: https://www.ebi.ac.uk/gwas/publications/31217584

## Human research participants

Policy information about studies involving human research participants and Sex and Gender in Research.

| | |
|---|---|
| Reporting on sex and gender | We obtained self-reported sex from the UK biobank and LifeLines cohort and confirmed this information by genotype data. We performed rank based inverse transformation on quantitative trait phenotypes within each sex to adjust for the difference in mean and variance between sex. Hence, our findings apply to both sexes. No individual level data including sex need to be reported in this study. The consent of using the data was approved by the data provider. |
| Population characteristics | UK Biobank is a large population-based cohort with nearly 500,000 participants, whose age ranged between 40 and 69 at recruitment, from 4 ancestries including European, East Asia, African, and South Asia. (see https://www.ukbiobank.ac.uk/key-documents/ for more details).<br>Lifelines is a large, multi generational cohort study from northern population of Netherlands. The cohort included participants from three generations. The participants' age ranged between 0 and 93 in this cohort.<br>The Biobank Japan (BBJ) is a cohort of patients diagnosed with any of the 47 target diseases who were enrolled in Japan. The average age was 62.7 years for men and 61.5 years for women at recruitment.<br>The FinnGen project is a continuous research initiative that utilizes samples sourced from a nationwide network of Finnish biobanks and digital healthcare data retrieved from national health registers. Isolated Finn populations that have undergone recent bottlenecks can harbor harmful alleles predisposing to disease at much higher frequencies than what is expected in larger and older outbred populations, due to heightened genetic drift. The participants age ranged between 0 and 108 in this cohort.<br>The Population Architecture using Genomics and Epidemiology (PAGE) study performed GWAS of 26 phenotypes in 49,839 non-European individuals. PAGE examined putative causal genetic variants across multi-ethnic and admixed populations: African Americans, Asian Americans, American Indians, European Americans, Hispanic Americans, and Native Hawaiians from four groups representing nine large U.S.-based cohorts. The distribution of covariates is unknown in some cohorts from the data available publicly (e.g. HCHS/SOL: aged 18-74 years; WHI: all women, 50-79 years of age; MEC: 45-75 years of 85 age at baseline). |
| Recruitment | Recruitment of the samples has been described in previous studies. We do not produce new data and have cited the previous work. |
| Ethics oversight | University of Queensland Human Research Ethics Committee B |

Note that full information on the approval of the study protocol must also be provided in the manuscript.

# Field-specific reporting

Please select the one below that is the best fit for your research. If you are not sure, read the appropriate sections before making your selection.

☒ Life sciences ☐ Behavioural & social sciences ☐ Ecological, evolutionary & environmental sciences

For a reference copy of the document with all sections, see nature.com/documents/nr-reporting-summary-flat.pdf

# Life sciences study design

All studies must disclose on these points even when the disclosure is negative.

| | |
|---|---|
| Sample size | The sample size for training was determined by the maximum number of samples from European ancestry with imputed genotype data in the UK Biobank and the summary-level data available from GIANT and Biobank Japan. After the quality control, the UK Biobank contains unrelated individuals of 4 ancestries, i.e., European ( N= 347,800), East Asian ( N=2,252), South Asian ( N=9,436) and African ( N=7,006) and the sample size for selected traits ranged from 110,334 to 360,503. The LifeLines cohort contains 11,842 samples of European ancestry after quality |

control. Summary-level data from the GIANT consortium (Yengo et al 2018) has the median per-SNP sample size of 704,810 for height and 688,632 for BMI. Summary data for diastolic blood pressure (Evangelou et al) had a sample size of 756,595, and type 2 diabetes (Xue et al) has a sample size of 62,892. The sample size in selected traits in Biobank Japan ranged from 82,810 to 165,056; the sample size for PAGE ranged from 28,534 to 49,781; sample size for FinnGen range from 163,394 to 342,439. We used those dataset as training or validation, which is the largest sample size current available. We also investigated the improvement in prediction accuracy of our method along with different sample sizes by down sampling in the UK Biobank, the prediction accuracy will increase along with more samples. Current data is sufficient to demonstrate the improved prediction accuracy.

**Data exclusions**

UK Biobank:
We removed SNPs with MAF < 0.01, Hardy-Weinberg Equilibrium test P < 10e-10, imputation info score < 0.6 in European samples. We used the GCTA software to remove the cryptic relatedness in the UKB based on the HapMap3 SNPs in each population. The samples were pruned by estimated relatedness larger than 0.05, keeping the unrelated samples only. We also removed the samples with mismatched sex information in phenotype and genotype, and samples withdrawn from the participation.
We removed the SNPs that are not in common among UKB, the annotation baseline model BaselineLD v2.2 and LifeLines cohort, resulting in 7,356,518 imputed common SNPs and 1,154,522 HapMap3 common SNPs. The 55 traits with relatively large sample size (N>110,000) were extracted from all 4 ancestries which were further pruned to 28 traits by absolute phenotypic correlation of 0.1. The traits with a mean prediction accuracy < 0.01 among methods were removed. The phenotypes with continuous values were filtered within the range of mean +/- 7SD.
LifeLines:
We removed SNPs with imputation info score < 0.3, MAF < 0.0001 and HWE < 1e-6. We removed the sample with age < 20 years old and removed samples with the phenotypic value (height and BMI) out of the range of mean +/- 5SD. We further removed the related samples and retained 11,842 unrelated samples.
Public data from GWAS meta-analysis for height, BMI, diastolic blood pressure, and type 2 diabetes:
We removed the SNPs with per-SNP sample size out the range of mean +/- 3 SD and the difference in allele frequency between GWAS and LD reference samples larger than 0.2. The summary data was further QCed by DENTIST to filter the SNPs with potential errors, removed the SNPs with P_DENTIST < 5e-8 and P_GWAS > 0.01.
BioBank Japan:
We matched the SNPs in the summary data from BBJ and UKB and removed SNPs with MAF < 0.005 in either population. After QC, 4,906,538 SNPs remained with functional annotations, of which 1,011,961 SNPs were in the HapMap3 panel.
PAGE:
We matched the SNPs in the summary data from PAGE and UKB and removed SNPs with MAF < 0.005 in either population. After QC, 6,064,174 SNPs remained, of which 1,131,955 SNPs were in the HapMap3 pane

**Replication**

We replicated the simulation 10 times in each scenario to evaluate the performance of our method. For real data analysis, we performed 10 cross validation to obtain the average prediction accuracy across validation folds. For cross biobank and cross ancestry prediction, no replication was performed because we used the whole Biobank or summary-level data to maximize the statistical power.

**Randomization**

Randomization for sample collection was not relevant to this study because we performed the analysis in publicly available data. We randomized in the simulation and cross validation in real data, with details described in the Methods section.

**Blinding**

Blinding was not relevant to this study, because we performed the analysis in publicly available data, We did not use any study design that required blinding, with the details described in the Methods section.

# Reporting for specific materials, systems and methods

We require information from authors about some types of materials, experimental systems and methods used in many studies. Here, indicate whether each material, system or method listed is relevant to your study. If you are not sure if a list item applies to your research, read the appropriate section before selecting a response.

## Materials & experimental systems

| n/a | Involved in the study |
|---|---|
| ☒ | ☐ Antibodies |
| ☒ | ☐ Eukaryotic cell lines |
| ☒ | ☐ Palaeontology and archaeology |
| ☒ | ☐ Animals and other organisms |
| ☒ | ☐ Clinical data |
| ☒ | ☐ Dual use research of concern |

## Methods

| n/a | Involved in the study |
|---|---|
| ☒ | ☐ ChIP-seq |
| ☒ | ☐ Flow cytometry |
| ☒ | ☐ MRI-based neuroimaging |
